# Multilevel effects of light on ribosome dynamics in chloroplasts program genome-wide and *psbA*-specific changes in translation

**DOI:** 10.1371/journal.pgen.1007555

**Published:** 2018-08-06

**Authors:** Prakitchai Chotewutmontri, Alice Barkan

**Affiliations:** Institute of Molecular Biology, University of Oregon, Eugene, OR, United States of America; University of Tennessee at Knoxville, UNITED STATES

## Abstract

Plants and algae adapt to fluctuating light conditions to optimize photosynthesis, minimize photodamage, and prioritize energy investments. Changes in the translation of chloroplast mRNAs are known to contribute to these adaptations, but the scope and magnitude of these responses are unclear. To clarify the phenomenology, we used ribosome profiling to analyze chloroplast translation in maize seedlings following dark-to-light and light-to-dark shifts. The results resolved several layers of regulation. (i) The *psbA* mRNA exhibits a dramatic gain of ribosomes within minutes after shifting plants to the light and reverts to low ribosome occupancy within one hour in the dark, correlating with the need to replace damaged PsbA in Photosystem II. (ii) Ribosome occupancy on all other chloroplast mRNAs remains similar to that at midday even after 12 hours in the dark. (iii) Analysis of ribosome dynamics in the presence of lincomycin revealed a global decrease in the translation elongation rate shortly after shifting plants to the dark. The pausing of chloroplast ribosomes at specific sites changed very little during these light-shift regimes. A similar but less comprehensive analysis in Arabidopsis gave similar results excepting a trend toward reduced ribosome occupancy at the end of the night. Our results show that all chloroplast mRNAs except *psbA* maintain similar ribosome occupancy following short-term light shifts, but are nonetheless translated at higher rates in the light due to a plastome-wide increase in elongation rate. A light-induced recruitment of ribosomes to *psbA* mRNA is superimposed on this global response, producing a rapid and massive increase in PsbA synthesis. These findings highlight the unique translational response of *psbA* in mature chloroplasts, clarify which steps in *psbA* translation are light-regulated in the context of Photosystem II repair, and provide a foundation on which to explore mechanisms underlying the *psbA-*specific and global effects of light on chloroplast translation.

## Author summary

Our experiments address the effects of light on protein synthesis within chloroplasts, whose ~80 genes are essential for photosynthesis and account for a large fraction of the protein synthesis in leaf tissue. Light is necessary for photosynthesis but it also triggers photo-oxidative damage. It is known that light-induced changes in chloroplast translation aid photosystem repair and prioritize energy consumption in the dark. However, prior studies have been limited in scope and offer conflicting views of the nature of these responses. In this study, we used new methods to generate a comprehensive description of chloroplast translation in maize at various time scales after shifting plants from dark to light and *vice versa*. We discovered that *psbA* mRNA, which encodes a protein that is particularly prone to photodamage, exhibits dynamic changes in ribosome occupancy in response to light and that it is unique in this regard. Ribosome occupancy on other chloroplast mRNAs is static even after many hours in the dark; however, these mRNAs are nonetheless translated at reduced rates in the dark due to a reduced rate of ribosome movement. Resolution of these superimposed effects clarifies the phenomenology of light-regulated chloroplast translation and provides a basis for exploring underlying mechanisms.

## Introduction

Energy from sunlight fuels life on earth through the process of photosynthesis. Light is both an essential resource and a source of stress for photosynthetic organisms, as it damages cellular structures through photo-oxidative processes and the production of reactive oxygen species. Photosynthesis is compromised when excess light damages the Photosystem II (PSII) reaction center or when excitation of Photosystem I (PSI) and PSII is unbalanced [1, 2]. A key target of photodamage is the D1 reaction center protein of PSII, which is encoded by the chloroplast *psbA* gene. In an elaborate repair cycle, damaged D1 is degraded and then replaced with newly synthesized D1 [3, 4]. To compensate for its high rate of turnover, D1 is the most rapidly synthesized protein in photosynthesizing cells. In plants and algae, light exerts rapid changes in D1 synthesis at the level of translation [5]. The phenomenon of light-regulated *psbA* translation has been intensively studied due to its central role in maintaining photosynthetic homeostasis and the ease of monitoring D1 synthesis. However, light-regulated translation in chloroplasts is not limited to the *psbA* mRNA [6]. This has been documented most thoroughly for the *rbcL* mRNA, whose translation initiation and elongation rates have been shown to change in response to light [7–12].

Despite a large body of literature on light-regulated chloroplast translation, major gaps remain in the characterization of the basic phenomenology: i.e. which genes respond to light at the translational level, with what kinetics, and at which step in translation. Studies have been limited to the few most rapidly synthesized proteins, so no information is available for the majority of the chloroplast’s ~80 protein-coding genes. In addition, many studies examined light responses in plants that had been grown for extended periods in the absence of light, at which point effects on chlorophyll synthesis and energy supply confound data interpretation. For example, an influential series of reports employed a de-etiolation regime in which barley seedlings were germinated and grown in the absence of light for many days. Illumination of these plants triggered the incorporation of radiolabeled amino acids into chlorophyll binding proteins in isolated chloroplasts, a result that was initially interpreted as light-induced translation [13]. However, subsequent experiments showed that much of this effect was due to stabilization of nascent apoproteins by chlorophyll, whose synthesis is induced by light [14, 15]. The degree to which regulated translation contributed to that phenomenon remains unclear. Further ambiguities arise from the fact that many studies assayed translation in isolated chloroplasts or etioplasts [5], whose energy and redox status may differ from those *in vivo*.

In this study, we revisit the phenomenon of light-regulated chloroplast translation using ribosome profiling [16], a method that was not available at the time of the work summarized above. Ribosome profiling provides a genome-wide, quantitative and high-resolution snapshot of ribosome occupancy in an intact organism at the time of harvest. To minimize effects of light on energy supply, we used young maize seedlings grown in diurnal cycles prior to depletion of their seed reserves, and we monitored ribosome occupancy shortly after shifting plants from the light to the dark, or *vice versa*. We distinguished effects of light on translation initiation and elongation rates by following the kinetics of ribosome occupancy after introducing lincomycin, which specifically inhibits ribosomes at the first few codons in an open reading frame (ORF). Our results show that transition from dark to light is accompanied by a global increase in the rate of translation elongation in chloroplasts, and that the rapid recruitment of ribosomes to the *psbA* mRNA is superimposed on this global response. Surprisingly, the abundance and positions of ribosomes on all chloroplast mRNAs other than *psbA* are maintained largely unchanged even after 12 hours in the dark, implying a balanced decrease in rates of initiation and elongation. This comprehensive analysis clarifies the phenomenology of light-regulated chloroplast translation and provides a basis for mechanistic hypotheses to be tested in future studies.

## Results

### *psbA* experiences large changes in ribosome occupancy in response to light-dark shifts in maize, and is the sole chloroplast mRNA to do so

**Fig 1:**
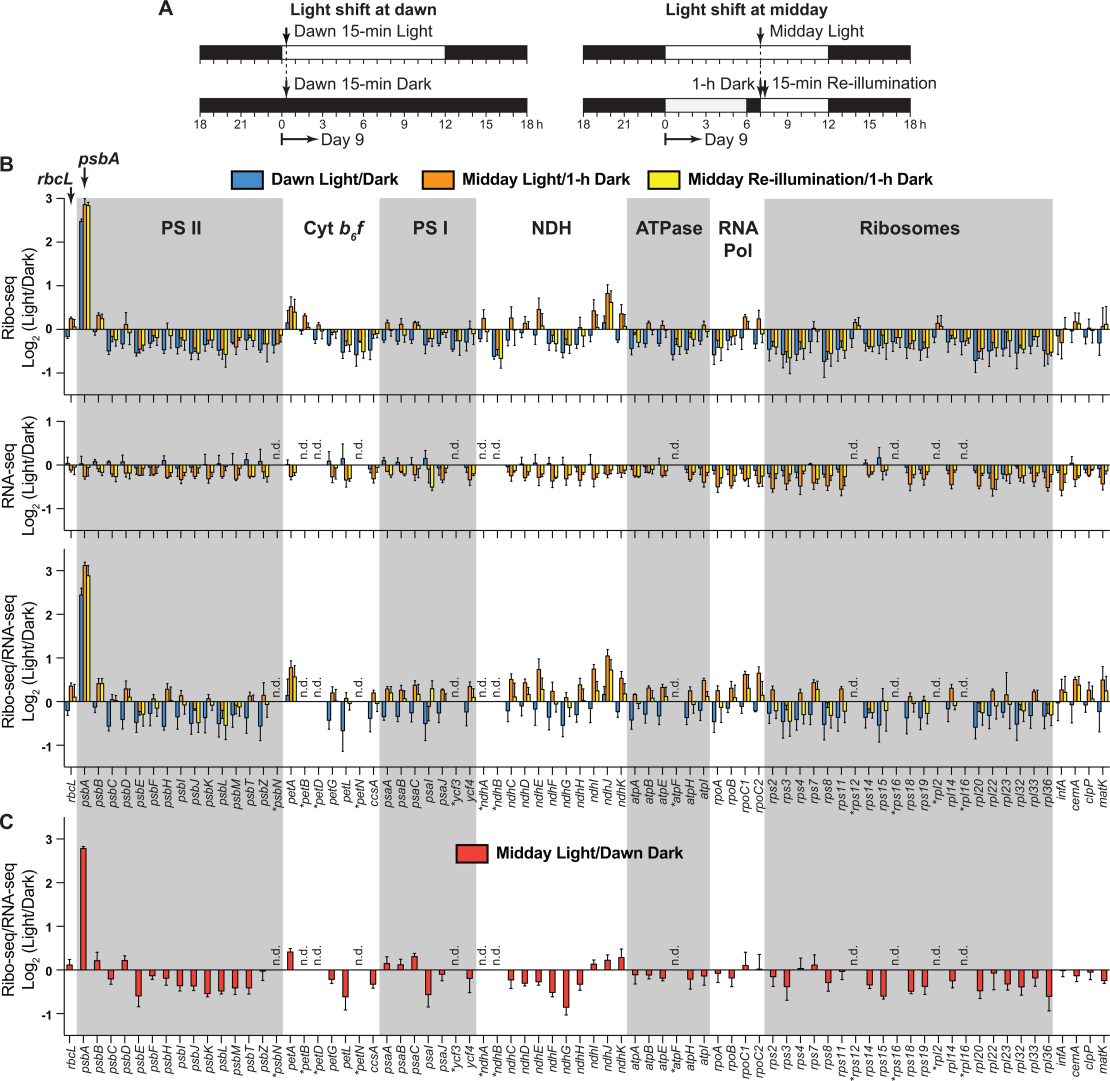
Genome-wide comparison of ribosome occupancy on maize chloroplast mRNAs following light-to-dark and dark-to-light shifts of green seedlings. (A) Light shift regimes used for this analysis. Seedlings were grown for 8 days in 12-h light/12-h dark cycles and subjected to the indicate light shifts on day 9. (B) Ratio of signal in the light to dark following short-term light shifts at dawn and midday. Values reflect the relative abundance of ribosome footprints (Ribo-seq) or RNA (RNA-seq) mapping to each ORF in light *versus* dark. Ribo-seq/RNA-seq values reflect the number of ribosomes per mRNA (ribosome density) in light *versus* dark. The values are the mean ± SEM from three replicate datasets. Genes are grouped according to their function rather than their genomic position. Genes for which mRNA levels could not be measured with confidence are marked with asterisks. These include very short ORFs and intron-containing genes, as explained in [17]. (C) Comparison of ribosome density on chloroplast ORFs after 12 hours in the dark (dawn) and 7 hours in the light (midday).

We grew maize seedlings for 8 days in light-dark cycles. Plants at this stage are photosynthetically competent but have not exhausted seed reserves. We subjected these plants to one of three light-shift regimes (Fig 1A): (i) Plants that experienced 15 minutes of light at dawn were compared with plants that were maintained in the dark and harvested at the same time; (ii) Plants that experienced one hour of dark at midday were compared with plants that were maintained in the light and harvested at the same time; (iii) Plants that were reilluminated for 15 minutes after one hour of dark at midday were compared with plants harvested prior to reillumination. We used a moderate light intensity (~300 μmol m^-2^s^-1^) to minimize photodamage. Three biological replicates were performed for each comparison.

Leaf tissue was processed for ribosome profiling (Ribo-seq) and RNA-seq analysis as described previously [17]. Ribo-seq reads mapping to the nuclear and chloroplast genomes exhibited the expected size distributions and three-nucleotide periodicity, and mapped primarily to protein-coding sequences (S1A–S1D Fig), demonstrating that they derive primarily from ribosome footprints. Read counts from chloroplast genes were normalized to the length of the ORF and sequencing depth by expressing them as reads per kilobase (in the ORF) per million reads mapped to nuclear protein coding sequences (RPKM). The Ribo-seq and RNA-seq RPKM values were highly reproducible across the replicates (Pearson Correlation ~ 0.99, S1E Fig). Every chloroplast gene was represented by at least 47 Ribo-seq reads and 1000 RNA-seq reads in each replicate, and the majority were represented much more deeply than this (S1F Fig).

The results are presented in Fig 1B as the ratio of values in light to dark for each of the three comparisons described above. The abundance of ribosome footprints on *psbA* RNA is highly dynamic in response to light, increasing approximately 6-fold after 15 minutes of light at dawn, decreasing approximately 8-fold after 1 hour in the dark at midday, and increasing approximately 8-fold after 15 minutes of reillumination at midday (see Ribo-seq, top panel). Strikingly, the *psbA* mRNA was the only chloroplast mRNA to behave in this manner: ribosome footprint abundance on all other chloroplast ORFs changed less than two-fold in each comparison. The abundance of *psbA* mRNA did not vary (see RNA-seq, middle panel), indicating that the changes in ribosome footprint abundance are due solely to effects on translation. Several other mRNAs showed small changes in ribosome density (ratio of Ribo-seq to RNA-seq reads) in response to light, but the change exceeded 2-fold only for *ndhJ* at midday (Fig 1B bottom). To assess whether additional differences become apparent at longer times after shifting from one condition to the other, ribosome density after 7 hours in the light (midday) is compared to that after 12 hours in the dark (dawn) in Fig 1C. This comparison shows that ribosome density on all chloroplast ORFs excepting *psbA* changes very little even after extended times in light or dark.

**Fig 2:**
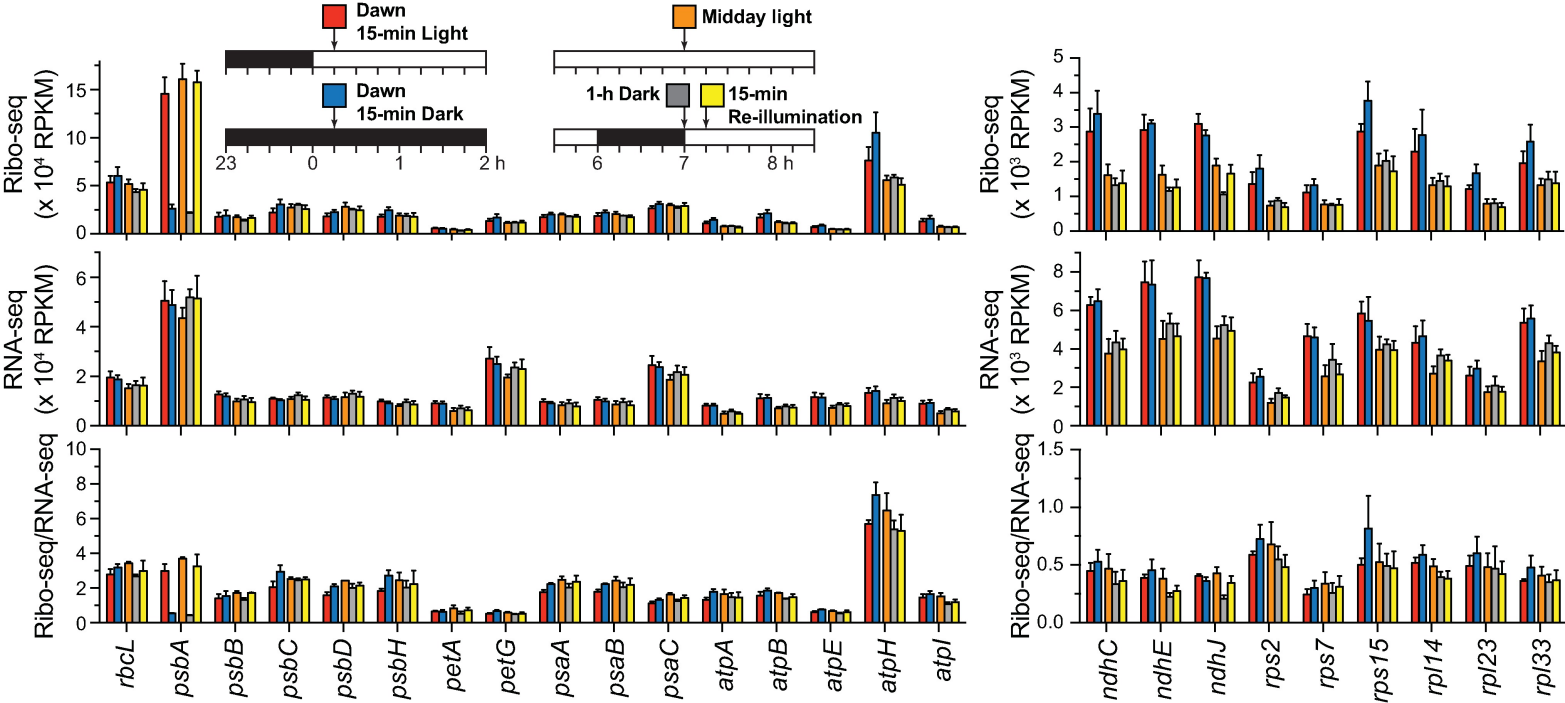
Ribosome footprint and RNA abundance for chloroplast genes following light shifts at dawn and midday. The values underlying the ratios presented in Fig 1 are displayed separately to allow comparison across all conditions. Analogous plots for the remaining chloroplast genes are shown in S2 Fig. As reported previously [17], the translational efficiency of *psbA* mRNA is not particularly high in the light (bottom panel, Ribo-seq/RNA-seq). Instead, the high translational output of *psbA* is due to the extremely high abundance of its mRNA (middle panel, RNA-seq).

Normalized read counts for each condition are plotted separately in Fig 2 and S2 Fig. This view of the data shows that mRNA abundance across all five conditions exhibited only small variations, although mRNAs from several genes were approximately 2-fold more abundant at dawn than at midday regardless of light condition. Furthermore, all chloroplast mRNAs other than *psbA* maintained similar ribosome density (Ribo-seq/RNA-seq) at all time points. The Ribo-seq RPKM from *psbA* after 1 or 12 hours in the dark was similar to that from other genes encoding photosystem subunits. Illuminating plants for 15 minutes restored *psbA* ribosome occupancy to the same level as that in plants that had been maintained in the light for 7 hours.

**Fig 3:**
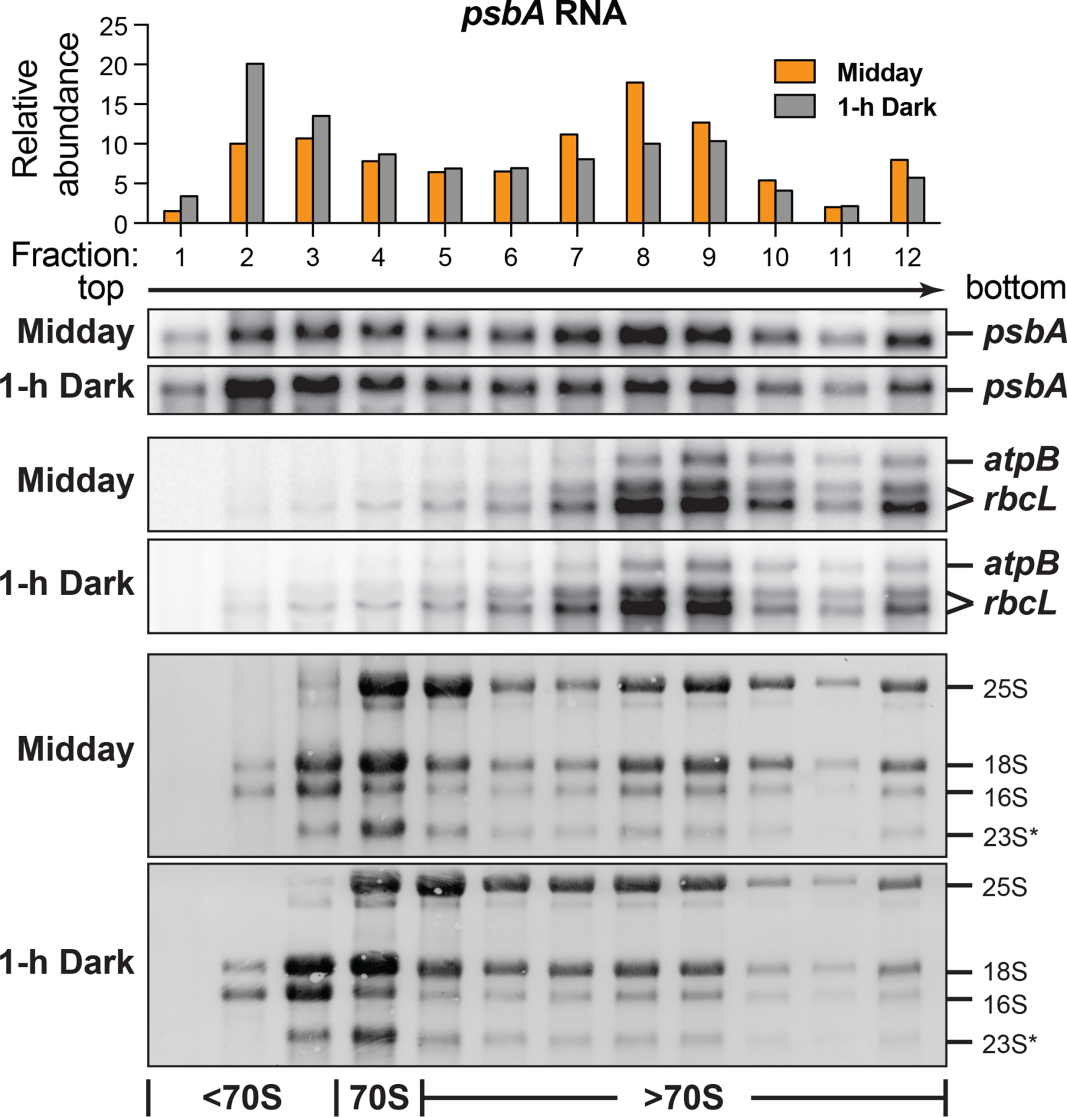
Polysome analyses support conclusions from Ribo-seq. Leaf lysates prepared from plants grown as for the ribosome profiling assays in Fig 1 were fractionated by sedimentation through sucrose gradients. RNA extracted from each fraction was analyzed by RNA gel blot hybridization. The blots shown here come from material harvested at midday, either in the light or after 1 hour in the dark. A single blot for each tissue sample was probed sequentially to detect *rbcL*, *atpB/E*, and *psbA* RNAs. The methylene blue-stained blots below illustrate the distribution of cytosolic (25S and 18S) and chloroplast (16S and 23S*) rRNAs in the same gradients. The quantification shown at top is based on quantitative phosphorimaging. S3 Fig shows the corresponding data for *ndhJ* and for material that was reilluminated for 15 minutes or harvested in the dark just prior to dawn.

These findings were corroborated by results from traditional polysome assays in which the association of mRNAs with ribosomes was assessed by their rate of sedimentation through a sucrose gradient. Consistent with the ribosome profiling data, the distribution of *psbA* mRNA shifted toward lower molecular weight fractions shortly after shifting to the dark (and *vice versa)*, whereas the sedimentation profiles of the *rbcL* and *atpB/E* mRNAs were indistinguishable even when comparing plants that had been in the dark or light for many hours (Fig 3 and S3 Fig). Although rapid sedimentation could result from the association of mRNAs with large non-ribosomal ribonucleoprotein particles, the ribosome profiling data argue against this possibility: the ribosome footprints exhibited similar size, 3-nucleotide periodicity and confinement to ORFs among all assayed conditions (S1 Fig). Together, these findings strongly support the interpretation that light has minimal impact on the ribosome-association of the vast majority of chloroplast mRNAs, whether comparing plants that have been shifted from one condition to the other for short (15 minutes) or long (many hours) periods of time. By contrast, cytosolic ribosomes shift toward smaller polysomes after 1-hour in the dark (Fig 3 bottom, 25S and 18S rRNAs), as reported previously for Arabidopsis [18].

### The distribution of ribosomes along chloroplast RNAs is similar in light and dark

**Fig 4:**
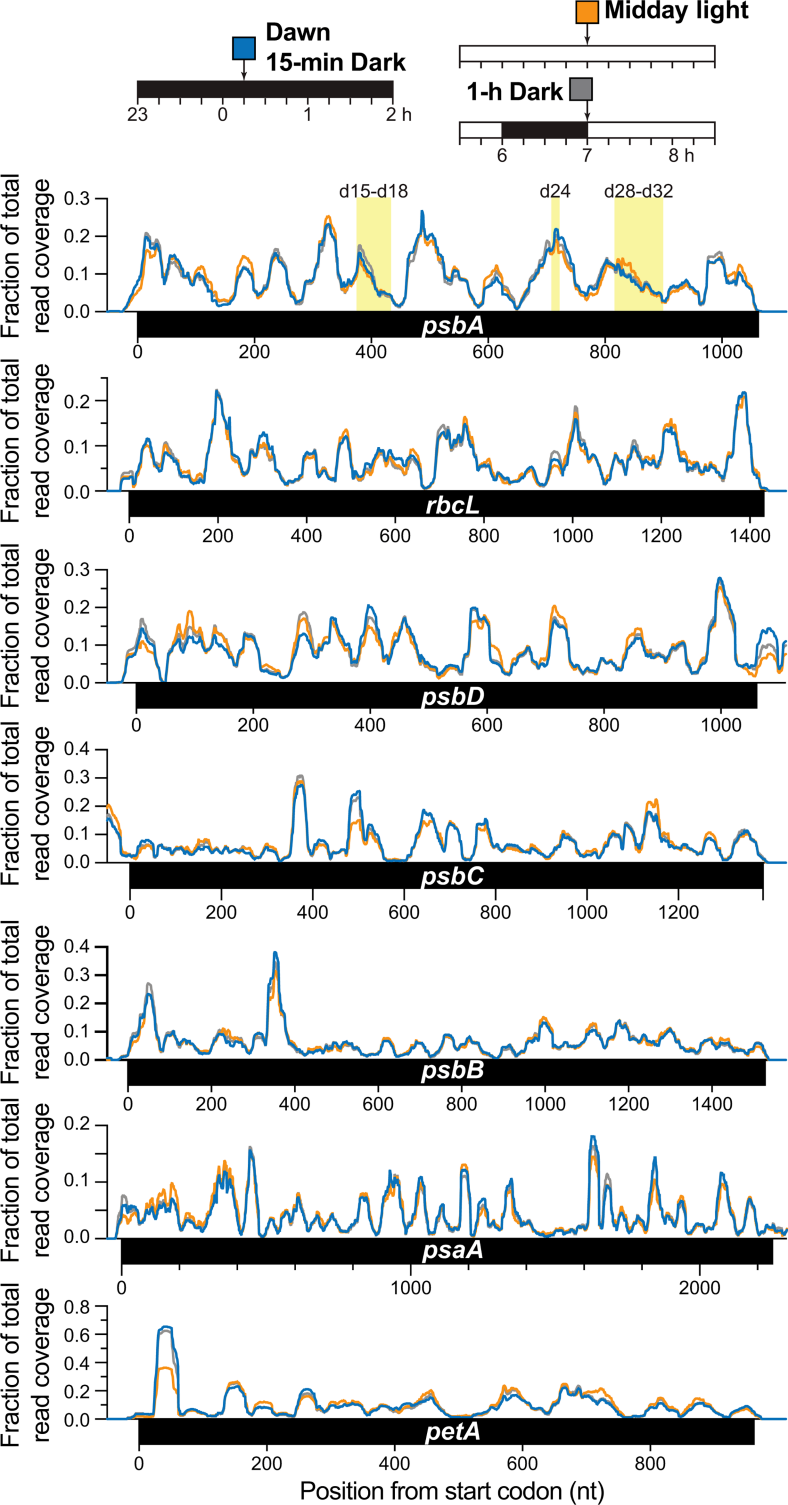
Distribution of ribosome footprints along chloroplast ORFs in light and dark. The distribution of Ribo-seq reads is plotted as a fraction of the total reads mapping to each indicated ORF for each of the three conditions diagrammed at top. Values are the sum from replicates 2 and 3. Replicate 1 was not included in this analysis because it involved a slightly different protocol for footprint and library preparation. Regions in *psbA* corresponding to major ribosome pause sites detected by toe-printing in barley are shaded in yellow and labeled as in the original reports [19, 20]. Ribosome footprints downstream of *psbD* and upstream of *psbC* result from the fact that these two ORFs overlap. A plastome-wide quantitative comparison of ribosome occupancy in light *versus* dark is presented in S4 Fig.

Pioneering studies detected paused ribosomes at several positions in the *psbA* RNA in isolated barley etioplasts, and showed that these pauses increase in magnitude after many hours of illumination [19, 20]. To assess the effects of light on ribosome pausing in developed chloroplasts, we examined the distribution of ribosome footprints along specific chloroplast ORFs; peaks in the distribution can be used to infer positions at which ribosomes dwell for an unusually long time [21]. Ribosome distribution along *psbA* was remarkably similar in material harvested at each assayed time point following a shift from one condition to the other (Fig 4), arguing against a role for regulated ribosome pausing in light-regulated *psbA* expression. Analogous plots for six other chloroplast ORFs are shown in Fig 4, and likewise showed very similar ribosome distributions in all conditions; that said, a site near the *petA* start codon appeared to be more highly occupied in the dark than in the light. A quantitative, plastome-wide comparison of ribosome distributions detected several locations at which ribosome dwell-time may differ to a small degree in dark and light (S4 Fig). Although several of these were reproducible in our experiments, their statistical significance is unclear. These possible exceptions aside, our results demonstrate that light does not cause wide-spread or high-magnitude changes in the pausing of ribosomes at specific locations on chloroplast mRNAs.

### Time course of changes in *psbA* ribosome occupancy and PsbA synthesis following light shifts

**Fig 5:**
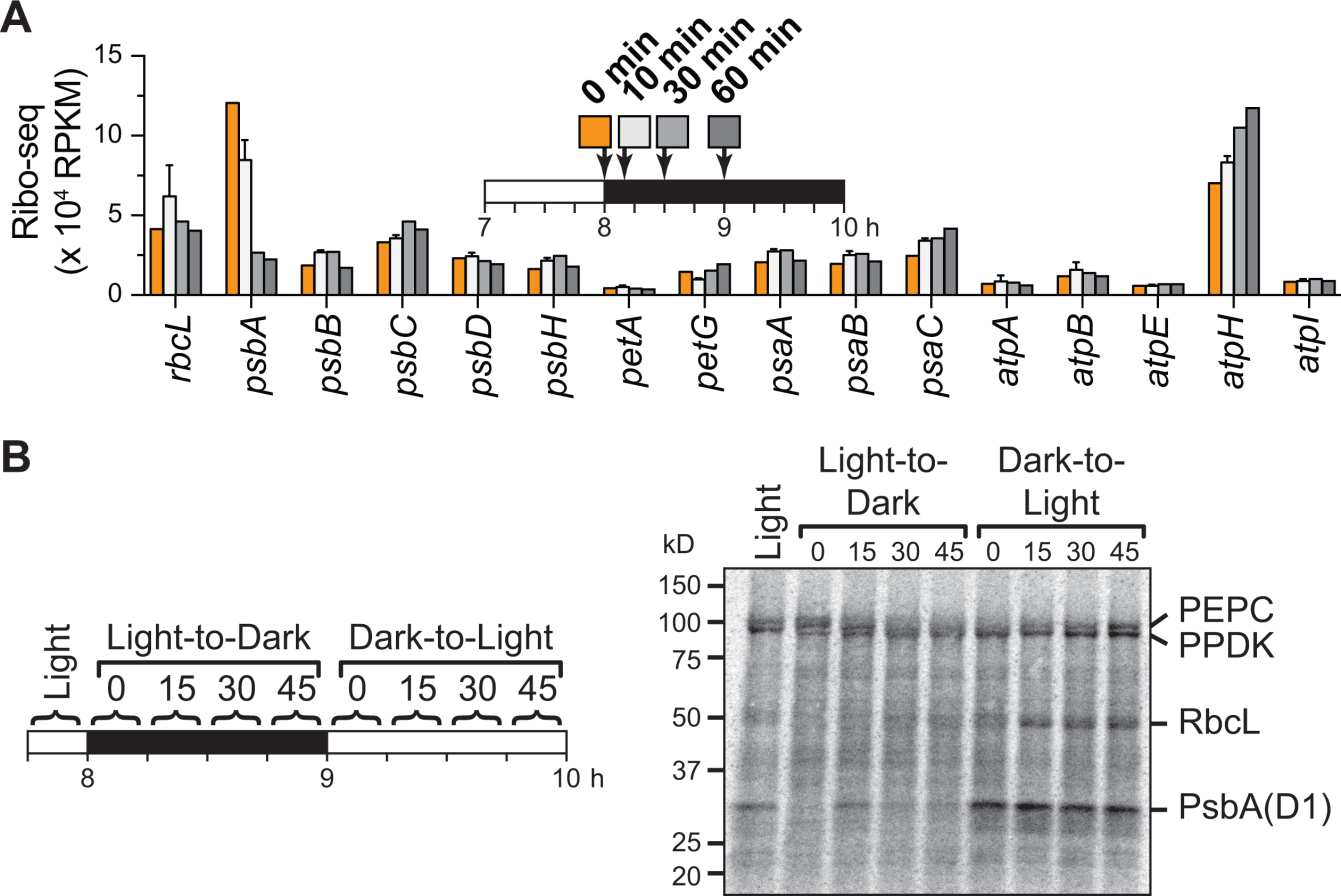
Time course of changes in *psbA* ribosome occupancy and protein synthesis following light shifts in maize seedlings. (A) Kinetics of ribosome loss on *psbA* following a shift to dark. Plants were processed for ribosome profiling at the indicated time points following a shift to the dark at midday. The 10-minute time point was performed in two replicates; the mean ± SEM is shown. Other time points were performed once. However, the 0 and 60 minute time points are equivalent to the triplicated data shown in preceding figures, which gave similar values. (B) *In vivo* pulse labeling following light-to-dark shifts. Labeling with ^35^S-methionine/cysteine was initiated at the indicated times and continued for 15 min. Total leaf lysates were analyzed by SDS-PAGE and phosphorimaging. The two rapidly synthesized proteins at ~100 kDa are likely to be the nuclear gene products PEP Carboxylase and PPDK.

To examine the kinetics with which ribosomes are lost from *psbA* mRNA upon a shift to dark, plants were processed for ribosome profiling over a time course after shifting plants to the dark at midday (Fig 5A). A small reduction in ribosome footprints was apparent after 10 minutes in the dark, whereas 30 minutes was sufficient (or nearly so) to reduce ribosome occupancy to that after 1 hour in the dark. These data, together with those shown in Fig 2, show that new steady-state ribosome occupancies on *psbA* RNA are established within approximately 15 minutes after shifting plants to the light and 30 minutes after shifting plants to the dark.

Although ribosome occupancy is typically a good proxy for relative rates of protein synthesis within a cell (or organelle) under any single condition [22, 23], the relationship between ribosome occupancy and protein synthesis becomes unpredictable when comparing different conditions due to possible differences in rates of translation elongation. In fact, classic studies reported a decrease in the translation elongation rate on the *psbA* and *rbcL* mRNAs after shifting isolated chloroplasts (*psbA*) or intact plants (*rbcL)* to the dark [8, 24–27]. To address this possibility in maize seedlings, we used *in vivo* pulse-labelling assays to examine how the rates of RbcL and PsbA synthesis change over time after shifting plants to the dark or light (Fig 5B). Pulse-labeling was performed during four consecutive 15-minute windows following shifts to dark and back to light at midday. The results show that PsbA synthesis drops rapidly after the shift to the dark and increases rapidly after reillumination, correlating in a general sense with the ribosome profiling data. Notably, however, the decrease in PsbA synthesis was apparent in the first 15 minutes after the shift to the dark (lane “0” in Fig 5B), preceding the clearance of ribosomes from its mRNA. Although labeled RbcL was poorly resolved in these experiments, the results suggest that RbcL synthesis decreased after the shift to dark and increased again after the shift to light, despite the unchanged association of its mRNA with ribosomes. These results suggest that the rate of ribosome movement along the *psbA* and *rbcL* mRNAs slows shortly after the shift to dark, and is restored shortly after the shift back to the light.

### The rate of ribosome run-off in the presence of lincomycin reveals a plastome-wide decrease in the rate of translation elongation following a shift to dark

Results above show that ribosome occupancy on all chloroplast ORFs other than *psbA* changes very little following shifts from light-to-dark or dark-to-light, even after many hours in the new condition. This is consistent with two scenarios: either light has minimal effect on their translation or it triggers concerted changes in rates of initiation and elongation such that the average number of ribosomes associated with each mRNA is maintained. The data shown in Fig 5 together with the prior studies discussed above support the view that the rate of ribosome movement along the *psbA* and *rbcL* mRNAs does decrease soon after a shift to the dark. However, it is not known whether other chloroplast mRNAs are similarly affected.

**Fig 6:**
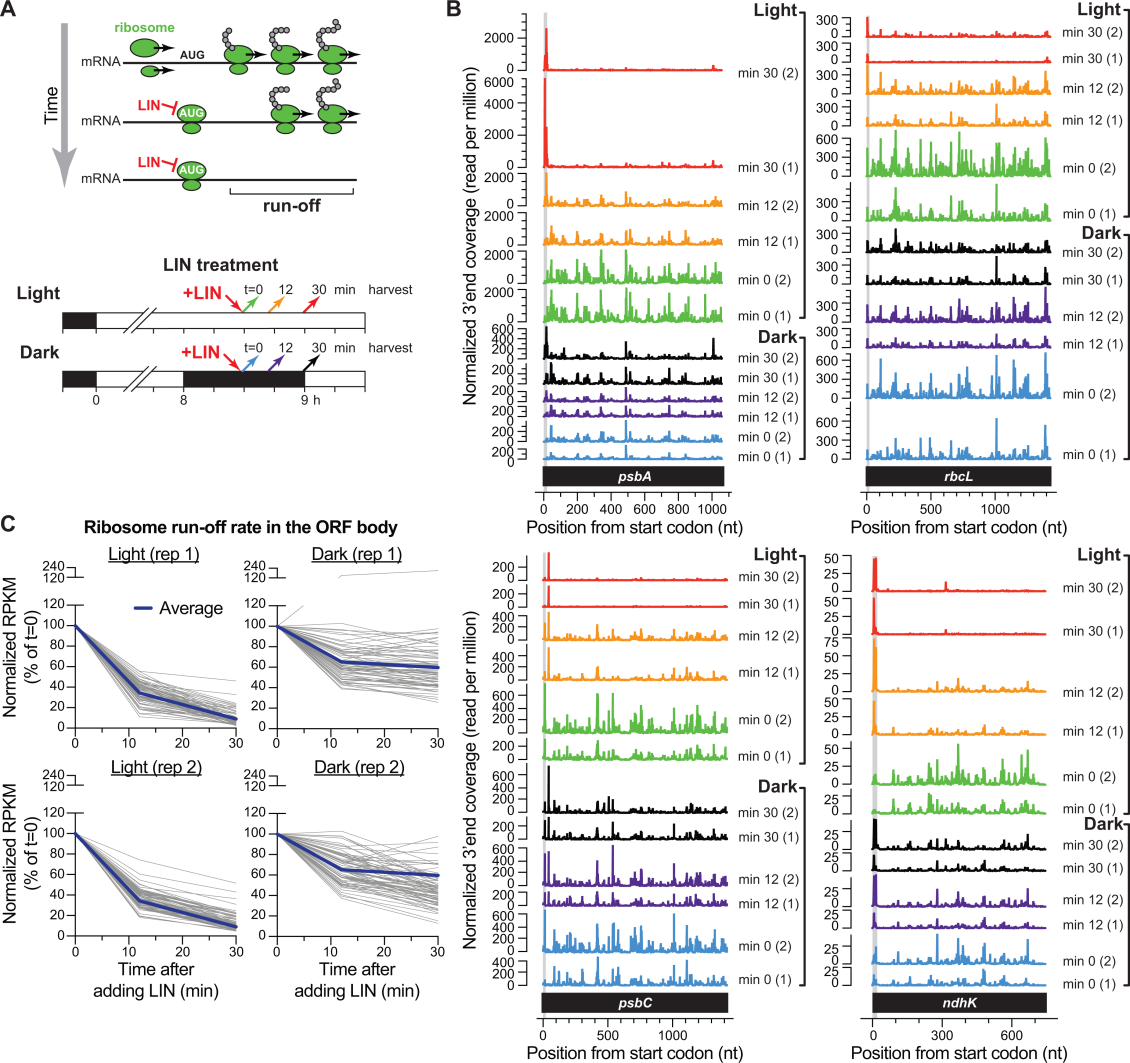
Time course of ribosome clearance following lincomycin treatment in light *versus* dark. (A) Experimental design. Lincomycin (LIN) inhibits peptide bond formation only when the nascent peptide is shorter than approximately five amino acids. Therefore, ribosomes with longer nascent peptides continue to elongate in its presence. LIN was applied to seedlings at midday in the light or after 30 minutes of dark adaptation; this period of dark adaptation is sufficient for ribosome occupancy on *psbA* mRNA to decrease to its dark steady-state level (see Fig 5). Material was harvested just prior to LIN treatment, and 12 or 30 minutes later. (B) Distribution of ribosomes along four chloroplast ORFs following LIN treatment in the dark or light. The plots show the normalized abundance of ribosome footprints with 3’ ends at each position. Plots for each of two replicates are shown separately to illustrate reproducibility. The region occupied by initiating ribosomes (first 7 codons) is shaded in gray. (C) Fractional change in ribosome footprint abundance on each chloroplast ORF following LIN treatment in the light and dark. The apical half of leaves 2 and 3 were processed for ribosome profiling. Each line shows the percent of the initial RPKM for a single chloroplast ORF over time following LIN treatment. The data from two replicate datasets are shown in separate plots to illustrate trends and variation. Footprints mapping to the first seven codons of each ORF were excluded from read counts because LIN inhibits these ribosomes. Ribosomes that build up at specific ORF-internal sites following LIN treatment (see Fig 7) were also excluded based on the criteria and rationale explained in Materials and Methods. Only genes whose average RPKM was greater than 100 at t = 0 are included (n = 70).

To provide a plastome-wide view of the effects of light on translation elongation, we performed ribosome profiling over a time course following treatment of seedlings with the peptidyl-transferase inhibitor lincomycin (Fig 6). Lincomycin does not inhibit ribosomes harboring nascent peptides longer than approximately five amino acids, so ribosomes that have passed the first few codons continue to elongate in its presence [28]. Thus, changes in the translation elongation rate will be reflected by changes in the rate of ribosome clearance from ORF bodies after treatment with lincomycin (see Fig 6A). Lincomycin does not inhibit cytosolic ribosomes, so we normalized chloroplast read counts to cytosolic read counts.

These experiments required that lincomycin be introduced into the chloroplasts of intact seedlings as rapidly as possible. Of the approaches we explored (see Materials and Methods), we found the introduction of lincomycin via thread wicks sewn through the stem to be most effective. Pilot experiments demonstrated that chloroplast protein synthesis in the leaf is inhibited starting approximately 10 minutes after initiating this treatment. Therefore, we harvested leaf tissue for ribosome profiling immediately prior to lincomycin treatment, and 12 and 30 min after initiating treatment (Fig 6A, bottom). For analysis of elongation rate in the dark, plants were dark-adapted for 30 minutes prior to lincomycin treatment, and were maintained in the dark throughout the treatment. During the 30-minutes of dark-adaptation, ribosome occupancy on *psbA* mRNA is reduced to its dark steady-state level (see Fig 5A); therefore, this experiment monitored the elongation rate only of those ribosomes that remained bound to *psbA* RNA after that time. We observed that the abundance and size distribution of ribosome footprints mapping to chloroplast start codons changed over time following lincomycin treatment (S5 Fig). The change in footprint size likely results from the fact that lincomycin traps ribosomes in a “rotated” conformation [29]. This effect was similar in the light and dark (S5 Fig), demonstrating that lincomycin inhibited chloroplast ribosomes in both conditions.

The distribution of ribosome footprints along the *rbcL* and *psbA* mRNAs at each time point following lincomycin treatment in the dark or light is displayed in Fig 6B (top). The rate of ribosome clearance is much slower in the dark than in the light, consistent with the prior evidence that light increases the rate of translation elongation on the *psbA* and *rbcL* mRNAs [8, 24–27]. Analogous plots for other genes show similar effects (Fig 6B bottom, S6A Fig), indicating that a reduction in translation elongation rate upon a shift to the dark is not specific for *psbA* and *rbcL*. We did not observe an obvious shift in ribosome footprints toward the 3’-end of ORFs over the lincomycin time course. This may be due to the fact that treatment of intact plants results in highly asynchronous exposure of chloroplasts to the antibiotic.

To provide a plastome-wide accounting of rates of translation elongation rate in light *versus* dark, ribosome footprint abundance on each ORF at each time point following lincomycin treatment is plotted as a percentage of the value just prior to lincomycin treatment in Fig 6C. The results show that the rate of ribosome clearance is, in general, considerably slower in the dark than in the light. A correlation plot of the ratio of ribosome footprints on each gene in light *versus* dark after 30 minutes of lincomycin treatment in the two replicates (S6C Fig) supports this conclusion, and suggests further that ORFs may differ in the degree to which the translation elongation rate drops following a shift to the dark. However, systematic differences between replicates suggest that varying transport of lincomycin to the apical region of the leaf accounts for some or all of this variation (see S6C and S6D Fig). Thus, additional experiments will be necessary to make firm conclusions about gene-specific differences in translation elongation rates.

We attempted to monitor the effects of light on translation initiation rates by following the buildup of ribosomes at start codons following lincomycin treatment. Ribosomes build up at the *psbA* start codon much more rapidly in the light than in the dark following lincomycin treatment (Fig 6B), consistent with the ribosome profiling and polysome data indicating that light stimulates the recruitment of ribosomes to the *psbA* mRNA. Furthermore, a metagene analysis showed that, on average, ribosomes build up at start codons more rapidly in the light than in the dark (S5 Fig). However, our ability to make inferences about initiation rates was complicated by several considerations. First, many chloroplast start codons did not accumulate ribosomes following lincomycin treatment either in the light or in the dark. Furthermore, the rate of ribosome build-up at start codons will be influenced by the reservoir of mRNAs with a vacant translation initiation region, a parameter that we cannot assess. That said, results above showing that (i) ribosome occupancy and distribution on most chloroplast ORFs is similar in the light and dark, (ii) the rate of ribosome movement along these ORFs is slower in the dark, and (iii) the average rate of ribosome buildup at start codons is greater in the light than the dark in lincomycin-treated plants, imply that the rate of translation initiation for most chloroplast mRNAs changes roughly in concert with changes in the elongation rate following light shifts.

### Ribosomes accumulate at ectopic sites resembling translation initiation regions during lincomycin treatment

**Fig 7:**
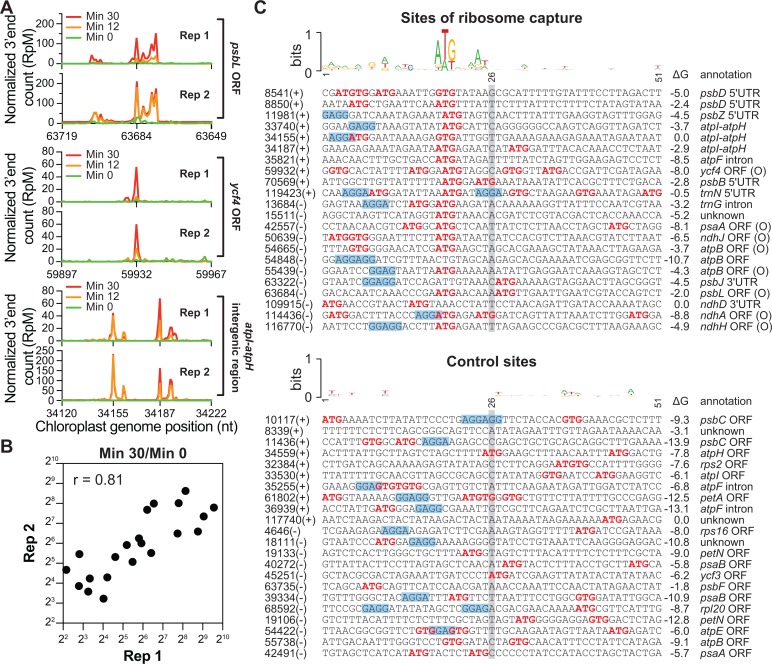
Ribosome capture at sites resembling translation initiation regions following lincomycin treatment. (A) Examples of ribosome build-up at non-start codons following LIN treatment. The abundance of ribosome footprints with 3’ ends mapping to the indicated position (per million reads mapped; RpM) is plotted at each time point following LIN treatment. Ribosome footprint abundance prior to LIN treatment is shown in green. Results from replicate experiments are shown in separate graphs. (B) Correlation plot showing the ratio of ribosome footprints 30 minutes after LIN treatment to that just prior to LIN treatment at non-start codons in two replicate experiments. These data come from the sites shown in panel (C), with the exception of the site at 8850, which was excluded because it had zero reads at t = 0 and was therefore unsuitable for the ratio calculation. (C) Features of sequences at which ribosomes accumulate to high-levels following LIN treatment. The sites of ribosome capture shown at top were selected based on the following criteria: they are not annotated start codons, they had an average of at least 50 RpM in the light, and the normalized abundance of the footprint increased at least 5-fold after 30 min in LIN. Sequences are centered at the 3’end of the footprint (gray shading). ATG and GTG sequences are in red. Sequences with four or more contiguous matches to the consensus Shine-Dalgarno sequence (AGGAGG) are shaded blue. Each sequence is annotated with its genomic coordinate and strand (left), and with the predicted stability of RNA structure (RNAfold, http://rna.tbi.univie.ac.at/cgi-bin/RNAWebSuite/RNAfold.cgi). Footprints are annotated “O” if they are out of frame with the ORF in which they reside. Each sequence at top is matched in an ordered pair to a control sequence below. The controls were selected as the sequence in closest proximity on the same strand but at least 500-nucleotide from each site that had a similar number of Ribo-seq reads at the start of lincomycin treatment in the light.

Unexpectedly, we observed that ribosome footprints accumulated to high levels at a number of sites outside of translation initiation regions over the lincomycin time course, even as bulk ribosomes cleared these genes as expected (see Fig 7A and S6B Fig for examples). The positions and magnitude of this feature were reproducible between replicate experiments (Fig 7B). The size-distribution of the footprints that build up at these sites was similar to that of lincomycin-bound ribosomes at start codons and distinct from that of elongating ribosomes (S5 Fig bottom), suggesting that these are footprints of ribosomes in the initiation mode. To examine that possibility, we compared the sequences around these “ribosome build-up” sites to those at sites from which ribosomes cleared following lincomycin treatment (Fig 7C). The sites of ribosome build-up (Fig 7C top) show a strong enrichment for sequences resembling ribosome binding sites: a start codon preceded by a predicted Shine-Dalgarno element. Several observations argue against the possibility that these are stalled elongating ribosomes: (i) the size distribution of their footprints resembles that of initiating ribosomes and not elongating ribosomes; (ii) many of them are out of frame with the ORF in which they reside and several are in UTRs; (iii) the abundance of ribosomes that accumulate at many of these sites exceeds that of the ribosomes found upstream prior to lincomycin treatment (see e.g. *psbL* in S6B Fig).

These observations strongly suggest that translation initiation complexes formed anew at ectopic sites following lincomycin treatment. This may be a consequence of the clearing of ribosomes from ORFs following lincomycin treatment, which will increase the accessibility of sequences resembling translation initiation regions while also increasing the concentration of free ribosomes available for initiation. Regardless of the mechanism, these footprints obscured calculations of ribosome run-off rates, and were therefore excluded from calculations of ribosome occupancy for the purpose of comparing elongation rates in light and dark (Fig 6C).

### Effects of light on chloroplast ribosome occupancy and protein synthesis in Arabidopsis

**Fig 8:**
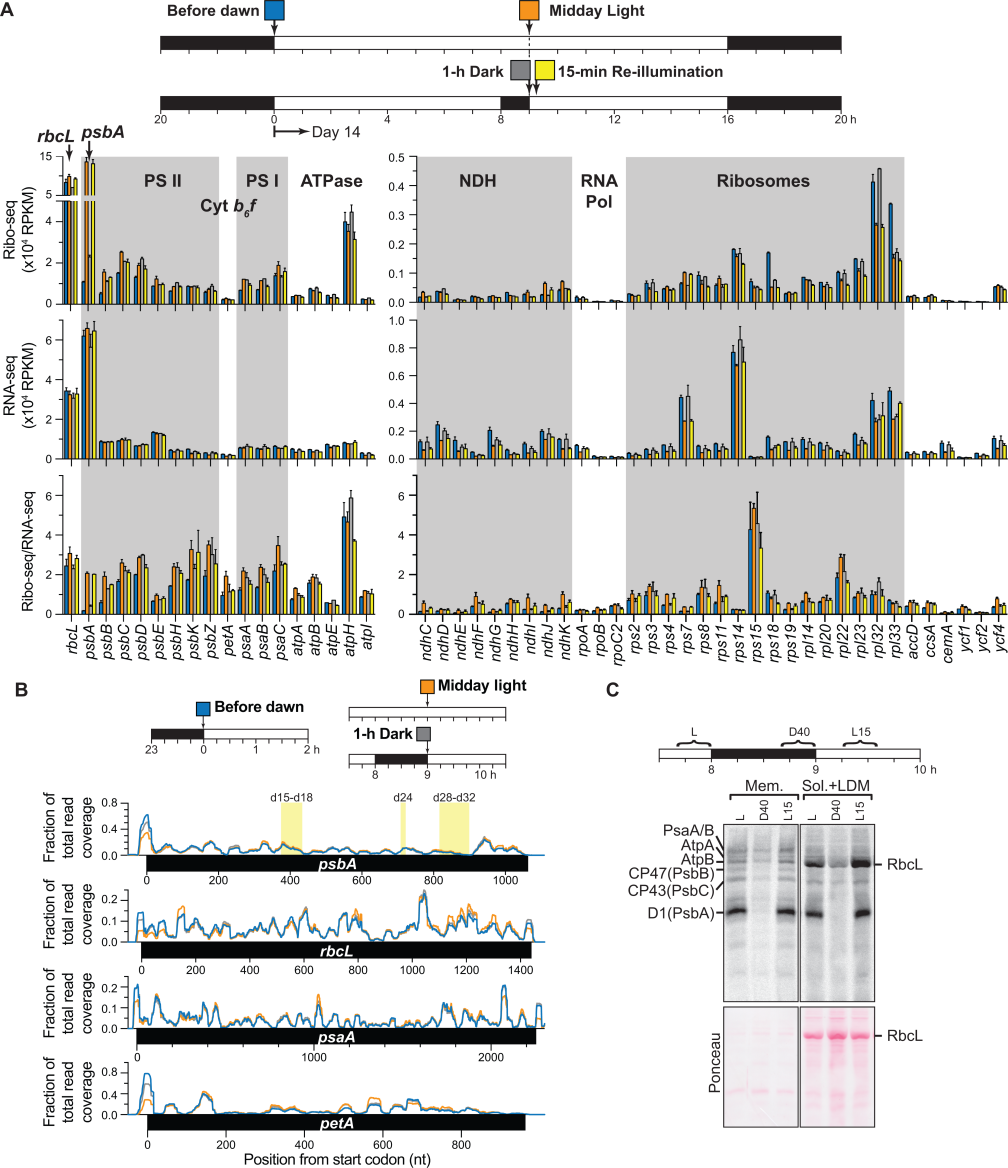
Chloroplast ribosome occupancy and protein synthesis in Arabidopsis following light-to-dark and dark-to-light shifts. (A) Seedlings were grown for 13-days in 12-h light/12-h dark cycles and subjected to the indicated light shifts on day 14. The normalized abundance of ribosome footprints (Ribo-seq), RNA (RNA-seq), or ribosome density (Ribo-seq/RNA-seq) is shown for each gene under each of the four indicated conditions. The values are the mean ± SEM from two biological replicates. Genes for which mRNA levels could not be measured with confidence are excluded. These include short ORFs (<150 nucleotides) and intron-containing genes. (B) Normalized read coverage for each of the three conditions diagrammed at top is plotted according to nucleotide position in each indicated ORF. Values are the sum from two replicates. Regions in *psbA* corresponding to major ribosome pause sites detected by toe-printing in barley are shaded in yellow and labeled as in the original report [19, 20]. (C) Chloroplast protein synthesis in Arabidopsis in response to light shifts at midday. ^35^S-methionine/cysteine was applied to excised seedling leaves in the presence of cycloheximide. Labeling was initiated at the indicated times and continued for 20 min. After separating dense membranes (Mem) from soluble and low-density membranes (Sol+LDM), proteins were resolved by SDS-PAGE, transferred to nitrocellulose, and detected by phosphorimaging. Bands corresponding to D1 (PsbA), CP47 (PsbB), CP43 (PsbC), PsaA/B, AtpA, AtpB, and RbcL are marked. The Ponceau S-stained nitrocellulose blots used for phosphorimaging are shown below. The recovery of radiolabeled D1 in the Sol+LDM fraction was likely due to inefficient pelleting of stromal lamellae, the site of PSII repair.

We repeated a subset of experiments with Arabidopsis to determine whether light affects chloroplast translation similarly in a C4 monocot (maize) and a C3 dicot (Arabidopsis) (Fig 8). Similar to maize, the *psbA* ORF in Arabidopsis showed a roughly 7-fold decrease in ribosome footprint abundance (Ribo-seq) and ribosome density (Ribo-seq/RNA-seq) following one hour in the dark at midday, and restoration to the original level after 15 minutes of reillumination (Fig 8A). Also as in maize, this highly dynamic response was unique to *psbA* and was not accompanied by a change in ribosome distribution (Fig 8B), implying that light did not have a substantive effect on the dwell time of ribosomes at specific sites. Several other genes showed a similar pattern of ribosome loss and gain but over a much smaller dynamic range (e.g. *ndhJ* and *ndhK*). In general, RNA levels were more dynamic in Arabidopsis than in maize over the conditions sampled. In particular, RNAs encoding many ribosomal proteins and NDH subunits increased after one hour in the dark and even more at the end of the night. For most ribosomal genes, this increase in RNA was reflected by an increase in ribosome footprint abundance such that ribosome density (Ribo-seq/RNA-seq) remained fairly constant. However, for most *ndh* genes, ribosome footprint abundance did not increase proportionally to RNA abundance in the dark, resulting in reduced ribosome density (see *ndhG*, *ndhI*, *ndhJ*, *ndhK)*.

The only striking difference between the data for Arabidopsis and maize involved ribosome density at the end of the night (dark blue bars in Fig 8A). Whereas in maize, ribosome density was maintained or even increased through the night for most chloroplast ORFs (Fig 1C, Fig 2), ribosome density was considerably lower at the end of the night than at midday for many genes in Arabidosis (e.g. *psbB*, *psbH*, *psbK*, *ndhC*, *rps11)*. Thus, although ribosomes remained associated with all RNAs through the night in both species, there was generally more clearing of ribosomes over the night in Arabidopsis.

To complement the ribosome profiling data, we used *in vivo* pulse-labeling to assay rates of chloroplast protein synthesis in Arabidopsis seedlings after shifting to the dark and following reillumination at midday (Fig 8C). These experiments were performed in the presence of the cytosolic translation inhibitor cycloheximide to facilitate the detection of chloroplast-encoded proteins. D1 synthesis decreased dramatically after ~40 minutes in the dark and was restored within ~15 minutes of reillumination, correlating with the change in ribosome footprint abundance on *psbA* RNA. The synthesis of all of the other identifiable proteins (RbcL, PsbB, PsbC, PsaA/B, AtpA, AtpB) also decreased following the dark shift and increased after reillumination, albeit with less dynamic range than D1 synthesis. Decreased synthesis of these proteins in the dark was not reflected by reduced ribosome footprint abundance (Fig 8A), implying that it results, at least in part, from a reduced rate of translation elongation.

Together, these results suggest that many of the themes established with the more comprehensive analyses in maize hold true also in Arabidopsis. That said, the data also suggest some differences that will be interesting to explore in the future. It remains to be determined whether these are meaningful differences or simply reflect differences in physiological status: Arabidopsis plants were grown on synthetic sucrose-containing medium, whereas maize seedlings were grown in soil and had not yet exhausted seed reserves.

## Discussion

Results presented here provide a plastome-wide accounting of ribosome occupancy, distribution, and elongation rate at various time scales following the transfer of maize seedlings with developed chloroplasts from dark to light, and *vice versa*. Our experiments resolved two layers of regulation: (i) ribosomes are gained and lost on the *psbA* mRNA shortly after shifting plants to the light or dark, respectively; (ii) the rate of translation elongation decreases globally following a shift to the dark. The superposition of a gene-specific recruitment of ribosomes to *psbA* on the global increase in translation elongation upon shifting from dark to light programs a massive increase in D1 synthesis. Ribosome occupancy and distribution on all other chloroplast ORFs changes very little, whether comparing short or long times (e.g. 15 minutes *versus* 7 hours) after the shift from one condition to another. That ribosome occupancy remains constant despite the global change in elongation rate suggests that initiation rates change roughly in concert with elongation rates on all chloroplast ORFs except *psbA*.

Most prior studies of the effects of light on chloroplast translation involved assays in isolated chloroplasts, and/or illumination of plants that had been germinated and grown in the absence of light and therefore lacked chlorophyll. These approaches provided important insights into the role of chlorophyll in stabilizing nascent chlorophyll binding proteins [30–32] and the impact of ATP and redox poise on *psbA* translation [26, 27, 33–35]. Prior studies showed further that translation elongation rates on several chloroplast ORFs decrease in the dark [8, 25, 26, 36, 37]. However, a coherent view of the effects of light on chloroplast translation has been lacking because (i) few genes (often only *psbA)* had been assayed, (ii) *psbA*-specific effects were rarely resolved from plastome-wide effects, (iii) *in organello* translation assays generally monitored only the elongation phase and employed ATP and reducing agents, which may have obscured light-induced effects that rely on fluctuations in these signals. By providing a comprehensive view of which chloroplast ORFs respond to light in plants harboring developed chloroplasts, over what time scales, and at what step in translation, our results provide a broad and relatively physiological context in which to interpret this large body of prior work. Our findings validate some prior views, expand on others, and suggest that some commonly held assumptions are incorrect.

### Light stimulates *psbA* translation initiation in the context of PSII homeostasis in plants

It is well known that light stimulates *psbA* translation in chloroplasts [5, 38, 39], so our finding that *psbA* ribosome occupancy changes rapidly when green plants are shifted between dark and light may appear to be nothing more than confirmatory. However, prior experiments involved illumination of undeveloped chloroplasts (de-etiolation) and/or failed to address whether light increases the rate of *psbA* translation initiation over and above any global stimulation. To our knowledge, the only prior studies to examine the effect of light on *psbA* ribosome association in plants involved the illumination of etiolated barley seedlings [9, 36, 40, 41]; in these experiments the *psbA* and *rbcL* mRNAs were recruited to polysomes following illumination, but this was mirrored by a global increase in chloroplast polysome content. A second set of experiments cited as evidence for light-induced *psbA* translation initiation employed a reporter gene fused to the *psbA* 5’UTR in tobacco plastids [42, 43]. However, these experiments compared reporter expression in etiolated seedlings to that after 24 hours (or more) of illumination, during which time the activation of photomorphogenetic programs might impact chloroplast gene expression. Furthermore, the possibility that increased reporter expression reflected a global increase in initiation and/or elongation rate was not addressed.

The regulation of *psbA* translation by light has been intensively studied in the single-celled alga *Chlamydomonas reinhardtii*. The prevailing view from this body of work is that *psbA* translation is regulated at different steps in the contexts of PSII biogenesis and repair; the former is believed to involve regulated initiation and the latter regulated elongation [5, 38, 44]. In our experiments, we exposed leaves with assembled photosystems to moderate light intensities. It is clear that the large and specific induction of *psbA* translation initiation we observed is a homeostatic repair mechanism: the response takes place in the context of developed chloroplasts, it is triggered within minutes of the shift to light, and it results in a substantial over-production of D1 with respect to other PSII subunits. Thus, our results add an important piece to this understanding by showing that light induces a rapid increase in the rate of translation initiation on the *psbA* RNA over and above any global increase in developed plant chloroplasts, and that this does not require excessive light intensities. The physiological and environmental contexts of light-regulated *psbA* translation are entirely different in land plants and Chlamydomonas: the former are sessile, multicellular, and land-dwelling whereas the latter are motile, single-celled, and aquatic. It would not be surprising if distinct mechanisms have evolved to maintain PSII homeostasis in these different contexts. That said, *psbA* mRNA in Chlamyomonas is lost from polysomes after one hour in the dark and regained within ~15 minutes of illumination at a moderate light intensity [45], much as we observed in maize and Arabidopsis. Although those experiments did not address whether this response was specific to *psbA*, they do suggest that light may regulate *psbA* translation at the initiation step in the context of PSII repair (and not just *de novo* biogenesis) in Chlamydomonas, as in plants.

### Light induces a plastome-wide increase in translation elongation rate but has little impact on site-specific ribosome pausing in plant chloroplasts

Classic studies demonstrated that the rate of translation elongation on several chloroplast mRNAs in plants changes in response to light. The two primary examples focused on (i) *psbA*, whose translation elongation rate decreased and increased when isolated chloroplasts were shifted to the dark and light, respectively [24–27, 46], and (ii) *rbcL*, which maintained polysome association after shifting Amaranth seedlings to the dark despite reduced RbcL synthesis [8]. By using ribosome profiling to follow the rate of ribosome run-off after treating maize seedlings with lincomycin, we were able to extend these conclusions in several ways. First, we showed that the effects on *psbA* translation elongation previously detected in isolated chloroplasts occurs also *in vivo*. Second, we showed that the effects on both *rbcL* and *psbA* are not gene-specific, but rather reflect a plastome-wide change in translation elongation rate. Our results leave open the possibility that the magnitude of this effect varies among ORFs, but additional experiments would be required to make firm conclusions in this regard.

Our results also clarified the effects of light on ribosome pausing. Pioneering ribosome toe-printing assays had revealed that ribosomes pause at specific sites on the *psbA* RNA in barley chloroplasts [19], leading to speculation that light may regulate *psbA* translation by altering pausing at specific sites. A subsequent study showed that toe-print patterns change many hours after illuminating etiolated barley seedlings [20]. By contrast, our data showed that light had no apparent impact on the distribution of ribosomes along the *psbA* mRNA in mature maize and Arabidopsis chloroplasts over the time scales we examined. Similar results were obtained for all other chloroplast ORFs. Thus, our results provide strong evidence that light does not have any major effects on ribosome pausing on chloroplast mRNAs. Although ribosome dwell time can be influenced by RNA structure, the sequence of the nascent peptide, and other features [21, 47], these behaviors do not appear to be modulated in the context of light-regulated protein synthesis in chloroplasts.

### Ribosome occupancy on chloroplast ORFs other than *psbA* is almost static in response to light shifts

Our finding that *psbA* was the only ORF to experience a substantial increase in ribosome occupancy following a transfer to light was unexpected because light has been reported to activate translation of several other chloroplast mRNAs via effects on proteins that bind their 5’-UTRs [38]. That ribosome occupancy on maize chloroplast mRNAs other than *psbA* was maintained almost unchanged even after twelve hours in the dark was also unexpected. Although there is prior evidence that ribosomes remain bound to several chloroplast mRNAs in the dark [8, 36], it has commonly been assumed that many chloroplast ORFs lose ribosome association over the course of the night; this is illustrated by proposals that special mechanisms are required to stabilize chloroplast RNAs at night due to their lack of ribosome association [e.g. 48]. Our results show that this is not the case in maize (excepting *psbA)*, and that it applies to no more than a handful of RNAs in Arabidopsis. Although we did not directly measure initiation rates, our measurements of ribosome occupancy and elongation rates suggest that ribosome occupancy is maintained through a concerted reduction in the translation elongation and initiation rates. The maintenance of ribosome association even after many hours in the dark may have evolved as a means to protect mRNAs from degradation, and to allow a rapid resumption of translation upon exposure to light.

### Mechanisms underlying the effects of light on chloroplast translation

Our results provide a foundation on which to delve into the mechanisms underlying each layer of light regulation, including the nature of the light-induced signal(s), the proteins whose activities are modulated by those signals, and the mechanisms by which these stimulate or inhibit translation in either a global or *psbA*-specific manner. The light-induced signals that trigger *psbA-*specific ribosome recruitment, the plastome-wide increase in elongation rate, and the plastome-wide increase in initiation rate may be shared or distinct. Studies involving isolated chloroplasts treated with inhibitors of photosynthetic electron transport [26, 34, 35, 37] have shown that light impacts chloroplast translation via its effect on photosynthesis. Photosynthesis-dependent changes in ATP, the ATP/ADP ratio, NADPH, reduced thioredoxin, reduced plastoquinone, stromal pH, and a *trans*-thylakoid proton gradient have all been invoked as potential triggers of light-induced effects on chloroplast translation [5]. Experiments that resolve the *psbA*-specific response from the global responses, and that assess the effects of disrupting specific steps in photosynthesis in intact plants will be required to pinpoint the photosynthesis-dependent signals that regulate chloroplast translation.

Changes in translation can be observed within minutes of shifting plants from light to dark (and *vice versa)*, strongly suggesting that the effects are mediated by post-translational modifications of pre-existing proteins. Candidates for proteins that regulate *psbA-*specific ribosome recruitment include HCF173 and HCF244, which are required specifically for *psbA* translation initiation in Arabidopsis [49, 50]. Plastome-wide effects may involve modifications of general elongation and initiation factors or other global translation modulators. For example, PSRP1, the chloroplast ortholog of a bacterial ribosome hibernation factor, has been proposed to repress chloroplast translation in the dark [51]. Future experiments can be directed toward testing these hypotheses, as well as assessing whether changes in light intensity and quality trigger behaviors similar to those described here. Given that the majority of chloroplast-encoded proteins reside in complexes that include nucleus-encoded subunits, our findings also raise the question of whether fluctuations in rates of chloroplast protein synthesis in response to light are reflected by corresponding changes in the synthesis of the nucleus-encoded proteins with which they partner.

## Materials and methods

### Plant material

*Zea mays* inbred line B73 was used for all experiments involving maize. Plants were grown in soil under light/dark cycles of 12 h/12 h (for experiments in Fig 1) or 16 h/8 h (for other experiments) at a temperature of 28° and 26°C for the light and dark periods, respectively. Plants were illuminated using a light intensity of 200–300 μmolm^-2^s^-1^, which is on the low end of typical “growth light” intensities used for maize and much lower than high-light treatments which typically exceed 1000 μmol m^-2^s^-1^. [e.g. 52, 53]. Light-shift experiments were performed on the ninth day after planting, at which time the third leaf was starting to emerge. The apical half of leaves two and three were used for ribosome profiling experiments. This tissue was flash-frozen in liquid N_2_ immediately after the indicated light treatment and stored at -80°C. Each Ribo-seq replicate pooled tissue from three seedlings. A single seedling was used for each pulse-labeling assay.

Arabidopsis (*Arabidopsis thaliana* Col-0) seedlings were grown on agar plates (1x Murashige and Skoog Basal Medium (Sigma), 0.3%(w/v) Phytogel, 1% (w/v) sucrose, pH 5.8). Seeds were planted with 1-cm spacing to minimize shading. Plants were grown at 22°C under light/dark cycles of 16 h/8 h for 14 days for ribosome profiling, or for 10 days for pulse labeling. Light intensity was approximately 100 μmol m^-2^s^-1^, consistent with typical Arabidopsis growth light intensities and much lower than high-light treatments [54]. For sequencing, aerial parts were harvested from approximately 25 seedlings per replicate, flash-frozen in liquid N_2_ and stored at -80°C. The treatment and harvest of maize and Arabidopsis in the dark were performed under a dim green light.

### Lincomycin treatment

We explored multiple methods to introduce lincomycin into maize. Watering intact seedlings with a lincomycin solution resulted in an unacceptably slow response time. Vacuum infiltration of detached leaves had secondary effects on chloroplast ribosome behavior, as revealed by Ribo-seq analysis of a mock-treated sample. Addition of lincomycin to maize protoplasts completely inhibited D1 synthesis within 10–15 min, but protoplast preparation is time consuming and is unsuitable for studies of dark to light transitions. We ultimately chose to introduce lincomycin into maize seedlings through thread wicks, because chloroplast protein synthesis in leaves was inhibited in 10–15 min (as assayed by pulse-labeling) and seedlings remained intact. Cotton threads (DMC crochet thread size 5) soaked in 1 mg/ml lincomycin (Sigma) were sewn four times through the stem of each seedling beneath the first (lowest) leaf. With 2 threads in each sewing, each wick consisted of 8 threads. The threads were cut at ~5 cm and the ends were placed in a 1.5 ml tube containing lincomycin. The apical half of leaves two and three were processed for ribosome profiling.

### *In vivo* pulse labeling

For maize, the labeling was performed as in [55]. In brief, an emery board was used to create two parallel scratches (1 cm apart) across the upper surface of leaf two, approximately 3-cm from the leaf tip. About 50 μCi of EasyTag Express ^35^S Protein Labeling Mix (PerkinElmer: ^35^S-methionine and cysteine; >1000 Ci/mmol, 11 mCi/mL) in 10 μL was added to the wounds. After 15-min of labeling, a 3-cm tissue section spanning the wounds was harvested and frozen in liquid N_2_. Plants were illuminated with a light intensity of 250 μmol m^-2^s^-1^ or maintained in the dark, as indicated. The tissue was homogenized in protein homogenization buffer (10 mM Tris-Cl pH 7.5, 10% glycerol, 5 mM EDTA, 2 mM EGTA, 40 mM β-mercaptoethanol, 2 μg/mL pepstatin, 2 μg/mL leupeptin, 2 mM phenylmethylsulfonyl fluoride). Lysates were fractionated by SDS-PAGE and transferred to nitrocellulose. Radiolabeled proteins were then detected with a Storm phosphorimager. Because the labeling efficiency of the leaf scratch method is variable, we used radiolabeled cytosolic proteins to normalize sample loading. This precluded the use of cycloheximide, which limited the number of chloroplast gene products we could resolve.

For each Arabidopsis sample, the first two rosette leaves from three seedlings were pooled and placed in a clear 24-well plastic plate, with care taken to avoid overlap of leaves. Leaves were pre-incubated for 30 min in 135 μl of labeling buffer containing cycloheximide (20 μg/mL cycloheximide, 1x Murashige and Skoog Basal Medium (Sigma), 1% (w/v) sucrose, pH 5.8). Labeling was then initiated by the addition of 15 μl of EasyTag Express ^35^S Protein Labeling Mix. Labeling was performed for 20 min under a light intensity of approximately 100 μmol m^-2^s^-1^ (or in the dark, as indicated). After labeling, leaves were washed once in the labeling buffer (lacking ^35^S) and then frozen in liquid N_2_. The tissue was homogenized as for maize. The membrane fraction was collected by centrifugation at 13,000 x g for 5 min and washed once with the homogenization buffer. Samples were fractionated in SDS-PAGE gels containing 6M urea (11% polyacrylamide), with loading normalized on the basis of equal chlorophyll (assayed as in [56]). Proteins were transferred to nitrocellulose and imaged with a Storm phosphorimager.

### Ribosome profiling and RNA-seq

With the exception of the first replicate of the experiment in Fig 1, we prepared ribosome footprints and total RNA according to the small-scale protocol described in [57], and purified footprints between approximately 20 and 40 nucleotides. For the first replicate of the experiment in Fig 1, we used the protocol described in [17], and purified footprints between approximately 20 and 35 nucleotides.

Ribo-seq libraries were prepared using the NEXTflex Small RNA Sequencing Kit v2 or v3 (Bioo Scientific) with additional steps described previously [17]. rDNA was depleted after first strand synthesis using biotinylated DNA oligonucleotides together with Dynabeads M-270 Streptavidin or MyOne Streptavidin C1 (ThermoFisher). Replicate 1 of the experiments in Fig 1 used the set of 47 biotinylated DNA oligonucleotides described in [17]. All other experiments used the oligonucleotides described in [57]. For RNA-seq, total RNA samples extracted from aliquots of the same lysates used for Ribo-seq were treated with TURBO DNase (ThermoFisher) followed by treatment with the Ribo-Zero rRNA Removal Kit (Plant Leaf) (Illumina). One hundred ng of the rRNA-depleted RNA was used for library construction using the NEXTflex Rapid Directional qRNA-Seq Kit (Bioo Scientific). The libraries were sequenced on a HiSeq 4000, HiSeq 2500 or NextSeq 500 instrument (Illumina), with read lengths of 50 to 100 nucleotides for Ribo-seq and 100 nucleotides for RNA-seq.

### Processing sequencing data

Read processing, alignment and analysis were performed according to the procedures described previously [17]. In brief, adapter sequences were trimmed using cutadapt [58]. Ribo-seq analyses used reads with lengths between 18 and 40 nucleotides. Read alignments were performed using Bowtie 2 with default parameters [59]. Reads were aligned sequentially to the following gene sets, with unaligned reads from each step used as input for the next alignment: (i) chloroplast tRNA and rRNA; (ii) chloroplast genome; (iii) mitochondrial tRNA and rRNA; (iv) mitochondrial genome; (v) nuclear tRNA and rRNA; (vi) nuclear genome. For metagene analysis, all protein coding sequence (CDS) coordinates from all transcript variants were combined to make a union CDS coordinate. Custom Perl scripts extracted mapping information using SAMtools [60]. The distribution of ribosome footprint lengths and RPKM for both the Ribo-seq and RNA-seq data were calculated based only on reads mapping to CDS regions. For chloroplast RPKM calculations, the reads mapping to the first 10 and the last 30 nucleotides of the CDS, which arise from initiating and terminating ribosomes, respectively, were excluded, and we defined the total number of mapped reads as the number mapping to nuclear CDS. For intron-containing chloroplast genes, Ribo-seq RPKM was calculated only from the CDS in the last exon, with the exception of *rps12*, where exon 2 was used. The rationale for these choices was discussed previously [17].

For the lincomycin assays, the ribosome run-off time course was determined from the RPKM values in the ORF body (from codon 8 to the stop codon). Because maize chloroplast ribosome footprints translating the same codon share a similar 3’-end position regardless of footprint size [17], the normalized abundance of footprint 3’-ends was used to determine sites at which ribosomes accumulate during lincomycin treatment. The 3’-end positions were extracted using SAMtools and normalized to million reads mapped to nuclear protein coding sequences. Sites at which the normalized 3’-end coverage increased more than 5-fold after 30-min of lincomycin treatment in the light (see e.g. S6B Fig) were removed from calculations used for the ribosome run-off analysis shown in Fig 6C and S6C and S6D Fig. Evidence discussed in the text strongly suggests that these result from internal binding of ribosomes in the initiation mode rather than stalled elongating ribosomes. Ribosome build-up sites located outside annotated translation initiation regions that had >50 reads per million after 30 min of lincomycin treatment in the light were reported in Fig 7.

Read mapping statistics and chloroplast RPKM values from all the experiments are provided in S1 Table.

### Polysome analyses

Polysome analyses were performed as described previously [55]. The *psbA*, *atpB/E*, *rbcL* and *ndhJ* probes used for RNA gel blots correspond to maize chloroplast genome nucleotide positions 295–1074, 54590–55790, 57036–57607 and 50535–51014, respectively.

### Data deposition

Illumina read sequences were deposited at the NCBI Sequence Read Archive with accession number SRP133508. Alignments of reads to the maize chloroplast genome used Genbank accession X86563. B73 RefGen_v3 assembly (maizegdb.org) was used for other genomes. The gene set from maize genome annotation 6a (phytozome.jgi.doe.gov) was reduced to the gene set annotated in 5b+ (60,211 transcripts) (gramene.org). For Arabidopsis, we used TAIR10 genome and annotation (arabidopsis.org).

## Supporting information

S1 Table Read mapping statistics and chloroplast RPKM values from Ribo-seq and RNA-seq analyses. (XLSX)

S1 Fig Characteristics of the maize Ribo-seq and RNA-seq data. A) Size distribution of ribosome footprints. Values are the mean ± SEM from all fifteen samples analyzed in Fig 1 (All), from the three replicates of samples harvested after 12 h in the dark just before dawn (Dawn Dark), and the three replicates harvested after 7h light at midday (Midday).
(B). Three-nucleotide periodicity of Ribo-seq data. The frame placements were inferred from the locations of the 5’ ends of the different footprint sizes at the start and stop codons. Values are the mean ± SEM, from the same samples described in (A).
(C) Confinement of Ribo-seq reads to ORFs in chloroplasts. The upper panel shows RNA-seq read coverage across the chloroplast *atpI-atpH-atpF-atpA* transcription unit. The lower panels show Ribo-seq reads mapping to the same genes.
(D) Metagene analysis of reads mapping near all cytosolic start and stop codons. Read coverage is quantified from the same datasets summarized in (A).
(E) Pearson correlation coefficients between each sample pair combination were calculated using RPKM values for each protein coding gene in the chloroplast genome. The correlation coefficients were used as the input for hierarchical clustering. The number following each sample name refers to the replicate.
(F) Histogram showing depth of sequencing data. The plot shows the frequency distribution of chloroplast genes according to read count in the indicated maize Ribo-seq (top) or RNA-seq (bottom) light-shift datasets (mean of 3 replicates).
(TIF)

S2 Fig Ribo-seq and RNA-seq RPKM values for chloroplast genes following shifts to the light or dark. The data are the same as those in Fig 1 but are displayed as values rather than ratios to allow comparison across time points. Analogous plots for the remaining chloroplast genes are shown in Fig 2.
(TIF)

S3 Fig Effects of light on polysome association of *psbA*, *rbcL*, *atpB/E*, and *ndhJ* RNAs. The experiment was performed as described in Fig 3.
(TIF)

S4 Fig Plastome-wide comparison of ribosome stalling in light *versus* dark. Ratios of normalized read coverage for each of the light-shift comparisons diagrammed in Fig 1 are plotted according to position in the maize chloroplast genome. Only one of the large inverted repeats is shown. Read coverage at each position was calculated as the fraction of the total reads mapping to that ORF. Data from replicates 2 and 3 are plotted separately. Replicate 1 was not included in this analysis because it involved a slightly different protocol for footprint and library preparation. Positions with low read coverage (<30 reads per million reads mapped to nuclear CDSs) were discarded. Nine regions in the genome showed >2-fold change between light and dark samples in at least 2 comparisons; these are marked with vertical dashed lines and bold gene names. Seven of these regions map near start codons, and may represent effects of light on initiation rate rather than ribosome pausing. Regions in *psbZ* and *psbH* locate in the stop codon and near sequences encoding the first transmembrane segment, respectively.
(TIF)

S5 Fig Characteristics of chloroplast ribosome footprints following lincomycin treatment. Size distribution of chloroplast ribosome footprints at start codon regions (first 7 codons), in ORF bodies (codon 8 to stop codon), and at non-start codon regions that captured ribosomes following lincomycin treatment. The mean ± SEM are shown from 2 replicates. The build-up of ribosomes at start codons over the lincomycin time course reflects the fact that lincomycin does not inhibit initiation but does inhibit clearance from start codons. For the analysis of ribosome build-up at non-start codon regions (bottom), we selected sites at which ribosome occupancy increased >5-fold in the light after 30 min of lincomycin treatment (87 sites).
(TIF)

S6 Fig Additional examples of ribosome distributions on chloroplast ORFs after lincomycin treatment. (A) Distribution of ribosomes along the indicated chloroplast ORFs following LIN treatment in the dark or light. The plots show the normalized abundance of ribosome footprints with 3’ ends at each position. Plots for each of two replicates are shown in separate graphs to illustrate reproducibility (replicate shown in parentheses). The region occupied by initiating ribosomes (first 7 codons) is shaded in gray.
(B) Examples of ORFs that accumulate ribosomes at specific sites during the LIN time course. The non-start codon positions at which ribosomes accumulated more than 5-fold after 30 min LIN treatment in light are marked with vertical dashed lines.
(C) Correlation plot showing the ratio of the normalized RPKM (as in Fig 6C) in dark versus light for each chloroplast ORF after 30 minutes of LIN treatment. Each symbol represents the data for one ORF. A ratio greater than 1 indicates a reduced rate of elongation in the dark.
(D) Correlation plot showing the normalized RPKM in dark after 30 minutes of LIN treatment. Each symbol represents the data for one ORF. A higher value indicates slower ribosome clearance from the ORF body. The clearance of ribosomes from ribosomal protein genes was similar in the two replicates, whereas the clearance from photosynthesis genes (especially from *psbA*) was slower in Replicate 1. This difference correlates with the regions of the leaf at which these genes are preferentially expressed: ribosomal protein genes peak in translational output in the basal region, photosynthesis genes peak in the apical region, and *psbA* is one of just a few genes whose output increases all the way to the leaf tip [17]. This correlation suggests that lincomycin inefficiently accessed the leaf tip in the first replicate of the dark treatment.
(TIF)
