# Correction: Multilevel effects of light on ribosome dynamics in chloroplasts program genome-wide and *psbA*-specific changes in translation

**DOI:** 10.1371/journal.pgen.1007907

**Published:** 2019-01-03

**Authors:** Prakitchai Chotewutmontri, Alice Barkan

S1 Table has an error in Section IV (Arabidopsis light-dark shifts, rows 322 to 399 of columns D to AI). The read count and RPKM values are incorrectly matched with the column labels. This error does not affect the graph of these data shown in Fig 8A, which shows the correct values. Please view the corrected S1 Table below.

## Supporting information

S1 Table Read mapping statistics and chloroplast RPKM values from Ribo-seq and RNA-seq analyses. (XLSX)
