# Supplementary figures and images for: Multilevel effects of light on ribosome dynamics in chloroplasts program genome-wide and psbA-specific changes in translation
Source: PLoS Genet. 2018 Aug 6;14(8):e1007555. doi: 10.1371/journal.pgen.1007555 (PMC6095610; doi:10.1371/journal.pgen.1007555)

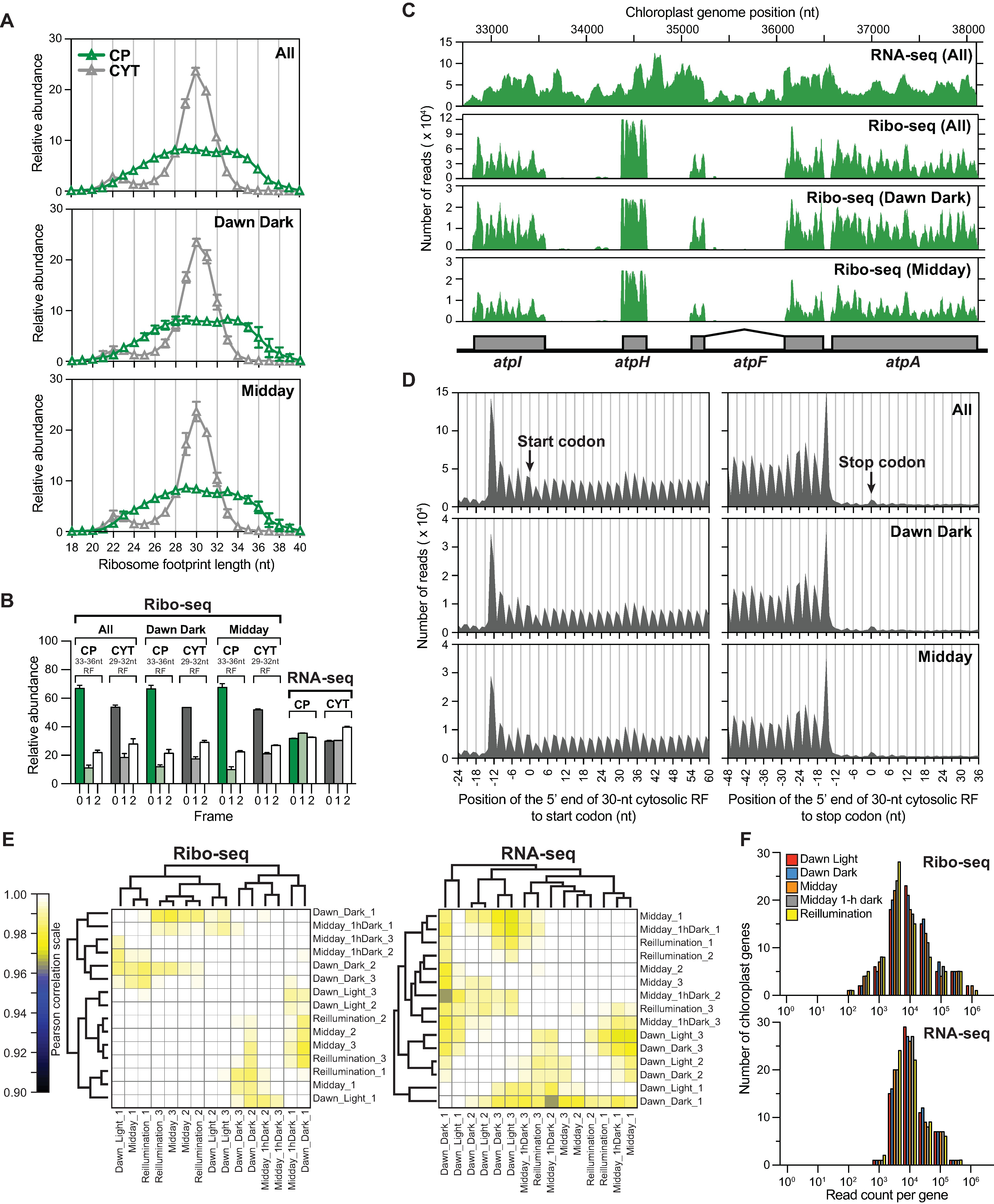

Supplement: S1 Fig — A) Size distribution of ribosome footprints. Values are the mean ± SEM from all fifteen samples analyzed in Fig 1 (All), from the three replicates of samples harvested after 12 h in the dark just before dawn (Dawn Dark), and the three replicates harvested after 7h light at midday (Midday). (B). Three-nucleotide periodicity of Ribo-seq data. The frame placements were inferred from the locations of the 5’ ends of the different footprint sizes at the start and stop codons. Values are the mean ± SEM, from the same samples described in (A). (C) Confinement of Ribo-seq reads to ORFs in chloroplasts. The upper panel shows RNA-seq read coverage across the chloroplast atpI-atpH-atpF-atpA transcription unit. The lower panels show Ribo-seq reads mapping to the same genes. (D) Metagene analysis of reads mapping near all cytosolic start and stop codons. Read coverage is quantified from the same datasets summarized in (A). (E) Pearson correlation coefficients between each sample pair combination were calculated using RPKM values for each protein coding gene in the chloroplast genome. The correlation coefficients were used as the input for hierarchical clustering. The number following each sample name refers to the replicate. (F) Histogram showing depth of sequencing data. The plot shows the frequency distribution of chloroplast genes according to read count in the indicated maize Ribo-seq (top) or RNA-seq (bottom) light-shift datasets (mean of 3 replicates). (TIF) [file pgen.1007555.s002.tif]

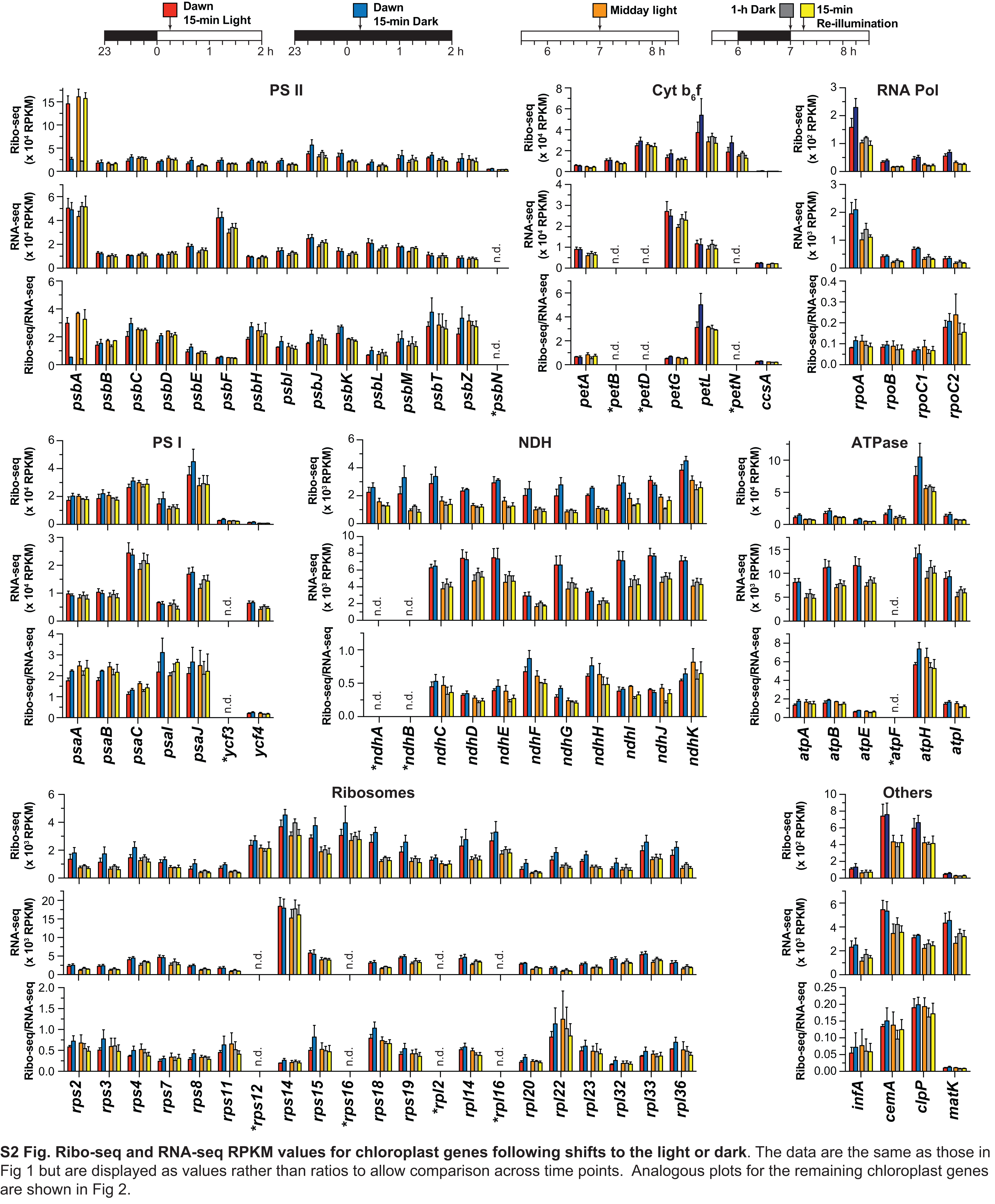

Supplement: S2 Fig — The data are the same as those in Fig 1 but are displayed as values rather than ratios to allow comparison across time points. Analogous plots for the remaining chloroplast genes are shown in Fig 2. (TIF) [file pgen.1007555.s003.tif]

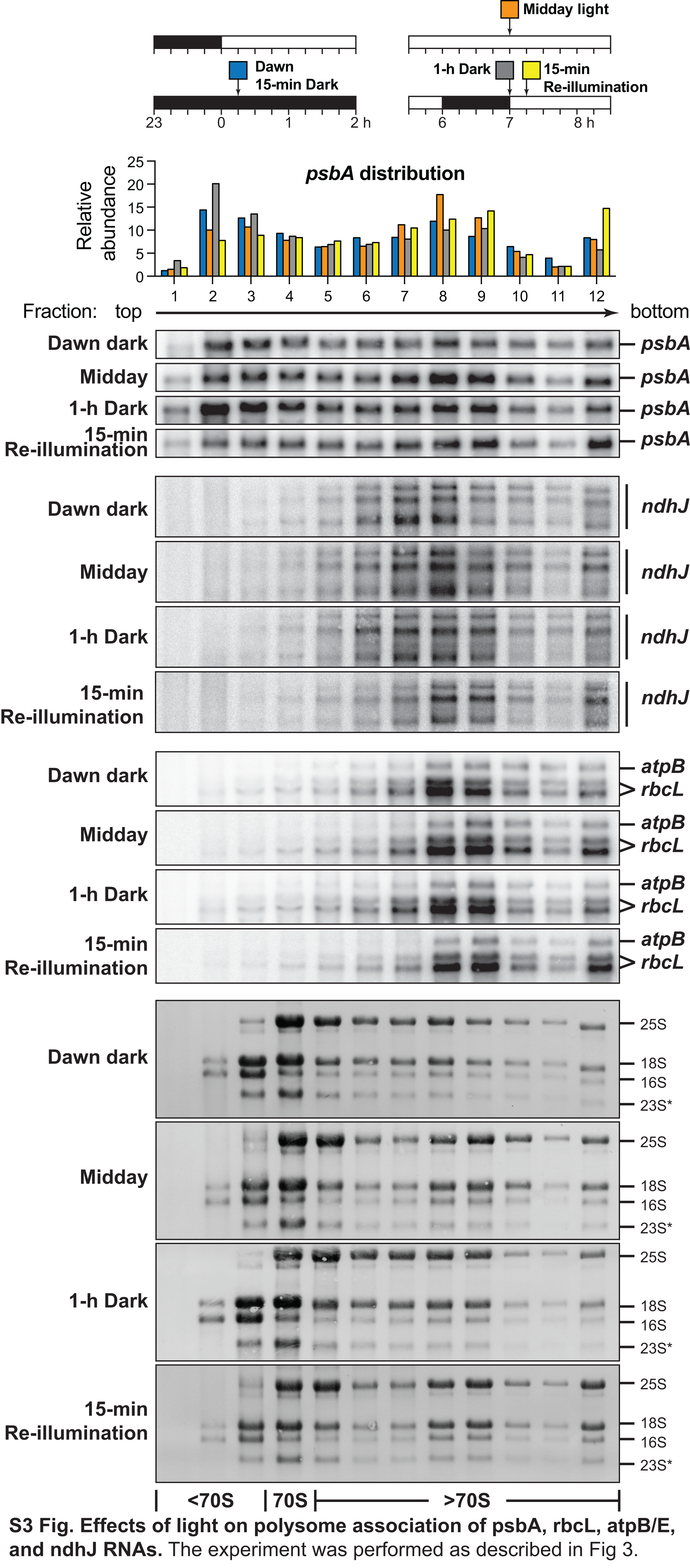

Supplement: S3 Fig — The experiment was performed as described in Fig 3. (TIF) [file pgen.1007555.s004.tif]

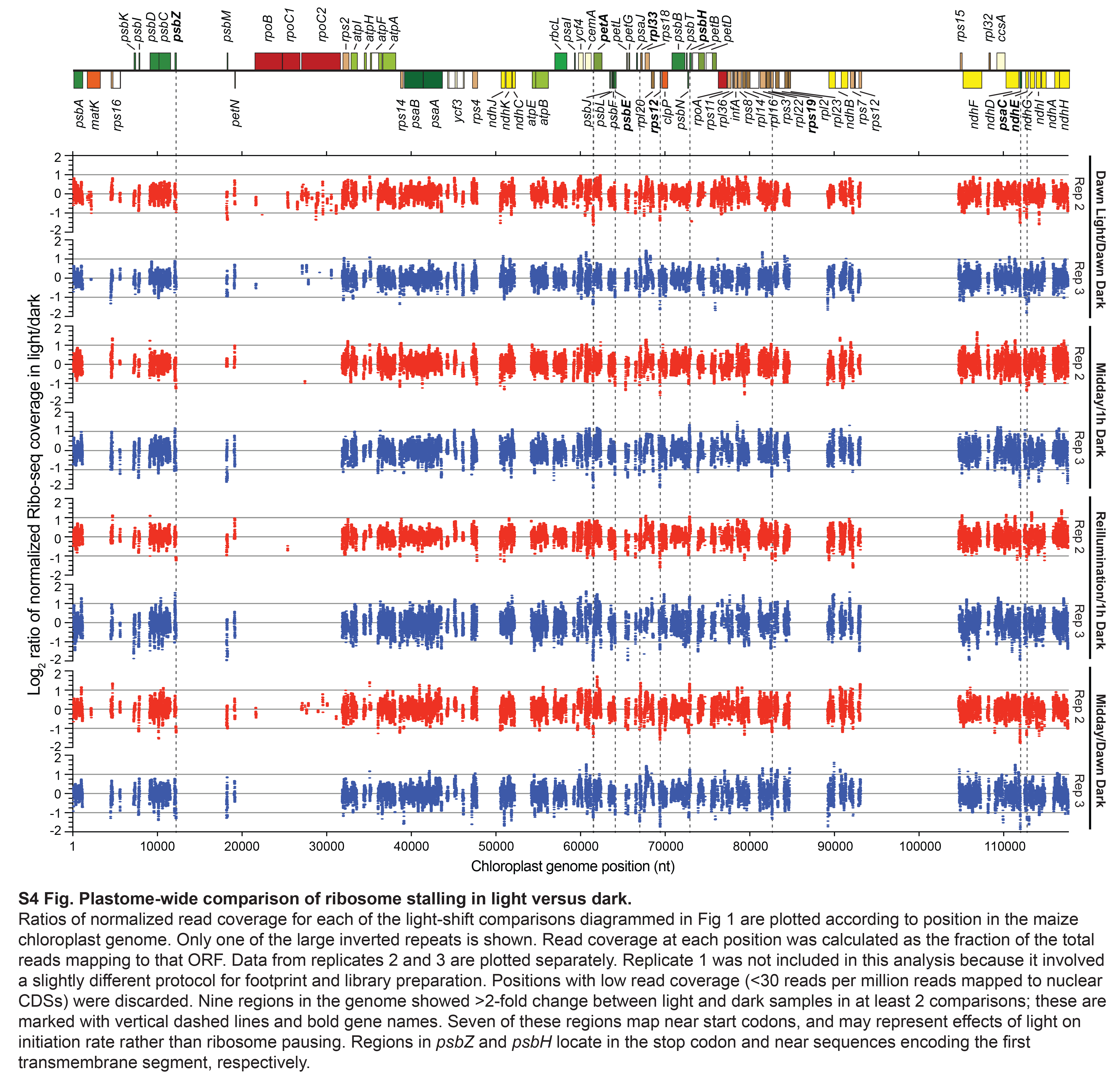

Supplement: S4 Fig — Ratios of normalized read coverage for each of the light-shift comparisons diagrammed in Fig 1 are plotted according to position in the maize chloroplast genome. Only one of the large inverted repeats is shown. Read coverage at each position was calculated as the fraction of the total reads mapping to that ORF. Data from replicates 2 and 3 are plotted separately. Replicate 1 was not included in this analysis because it involved a slightly different protocol for footprint and library preparation. Positions with low read coverage (<30 reads per million reads mapped to nuclear CDSs) were discarded. Nine regions in the genome showed >2-fold change between light and dark samples in at least 2 comparisons; these are marked with vertical dashed lines and bold gene names. Seven of these regions map near start codons, and may represent effects of light on initiation rate rather than ribosome pausing. Regions in psbZ and psbH locate in the stop codon and near sequences encoding the first transmembrane segment, respectively. (TIF) [file pgen.1007555.s005.tif]

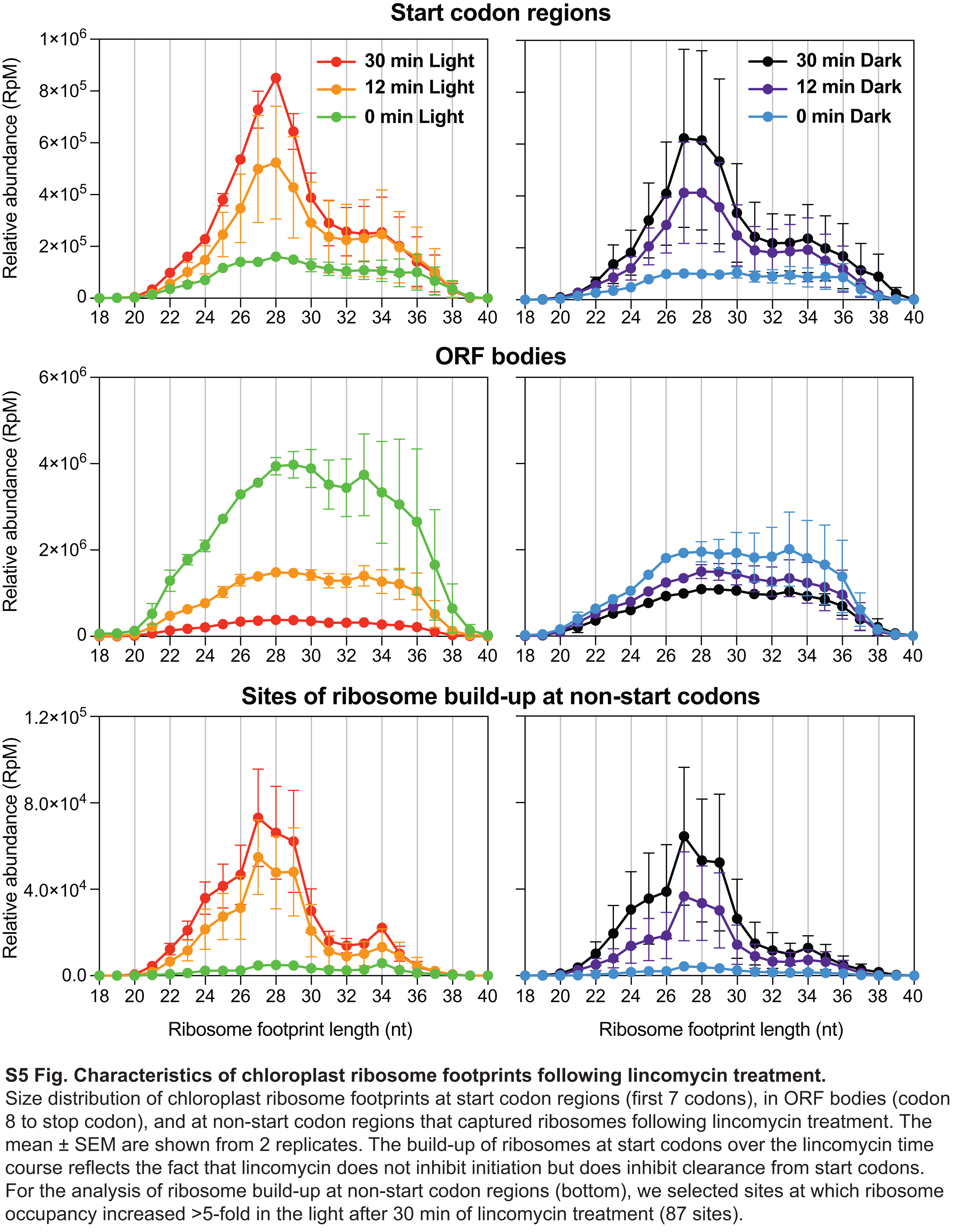

Supplement: S5 Fig — Size distribution of chloroplast ribosome footprints at start codon regions (first 7 codons), in ORF bodies (codon 8 to stop codon), and at non-start codon regions that captured ribosomes following lincomycin treatment. The mean ± SEM are shown from 2 replicates. The build-up of ribosomes at start codons over the lincomycin time course reflects the fact that lincomycin does not inhibit initiation but does inhibit clearance from start codons. For the analysis of ribosome build-up at non-start codon regions (bottom), we selected sites at which ribosome occupancy increased >5-fold in the light after 30 min of lincomycin treatment (87 sites). (TIF) [file pgen.1007555.s006.tif]

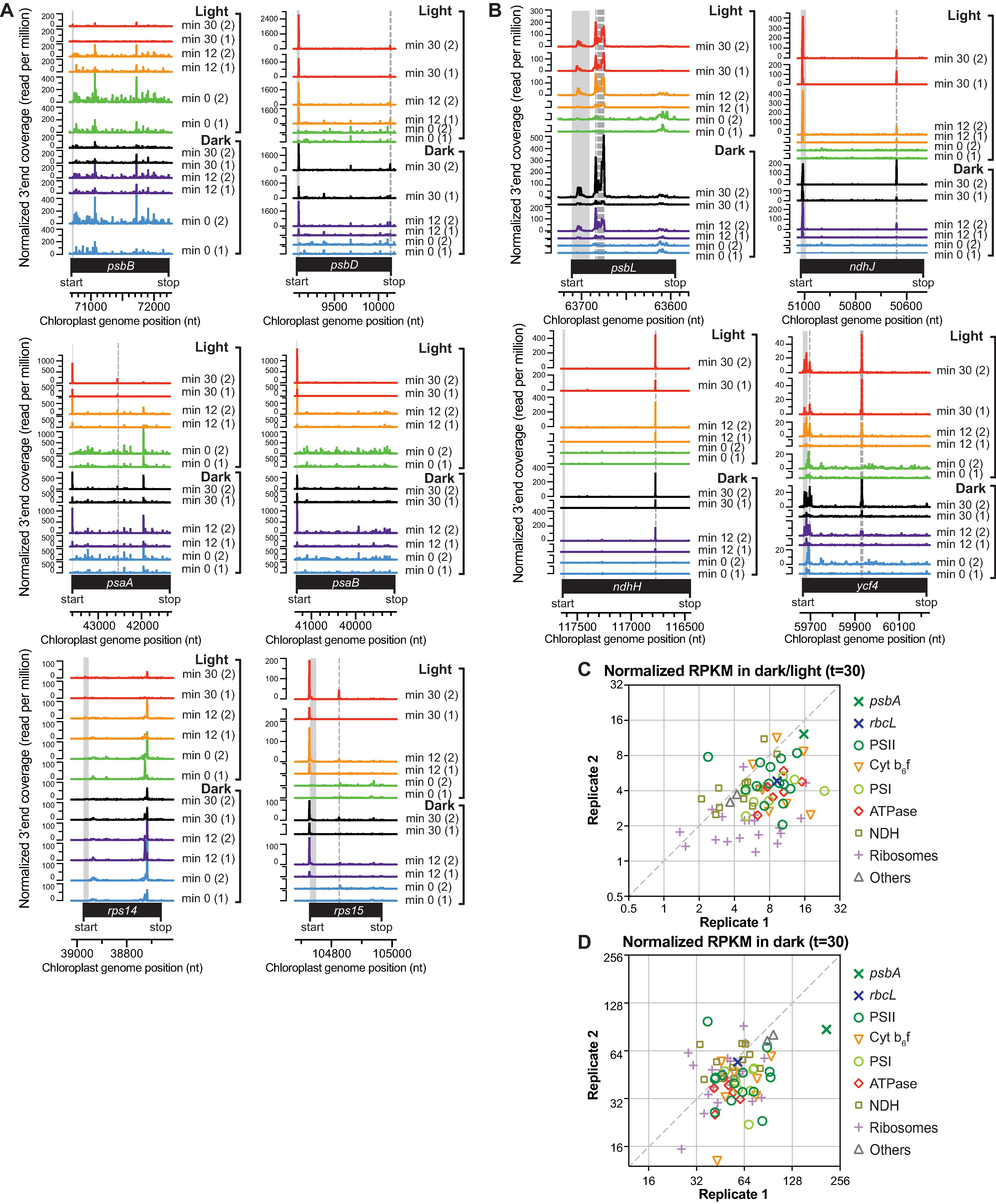

Supplement: S6 Fig — (A) Distribution of ribosomes along the indicated chloroplast ORFs following LIN treatment in the dark or light. The plots show the normalized abundance of ribosome footprints with 3’ ends at each position. Plots for each of two replicates are shown in separate graphs to illustrate reproducibility (replicate shown in parentheses). The region occupied by initiating ribosomes (first 7 codons) is shaded in gray. (B) Examples of ORFs that accumulate ribosomes at specific sites during the LIN time course. The non-start codon positions at which ribosomes accumulated more than 5-fold after 30 min LIN treatment in light are marked with vertical dashed lines. (C) Correlation plot showing the ratio of the normalized RPKM (as in Fig 6C) in dark versus light for each chloroplast ORF after 30 minutes of LIN treatment. Each symbol represents the data for one ORF. A ratio greater than 1 indicates a reduced rate of elongation in the dark. (D) Correlation plot showing the normalized RPKM in dark after 30 minutes of LIN treatment. Each symbol represents the data for one ORF. A higher value indicates slower ribosome clearance from the ORF body. The clearance of ribosomes from ribosomal protein genes was similar in the two replicates, whereas the clearance from photosynthesis genes (especially from psbA) was slower in Replicate 1. This difference correlates with the regions of the leaf at which these genes are preferentially expressed: ribosomal protein genes peak in translational output in the basal region, photosynthesis genes peak in the apical region, and psbA is one of just a few genes whose output increases all the way to the leaf tip [17]. This correlation suggests that lincomycin inefficiently accessed the leaf tip in the first replicate of the dark treatment. (TIF) [file pgen.1007555.s007.tif]
